# Serine-/Cysteine-Based sp^2^-Iminoglycolipids as Novel TLR4 Agonists: Evaluation of Their Adjuvancy and Immunotherapeutic Properties in a Murine Model of Asthma

**DOI:** 10.1021/acs.jmedchem.2c01948

**Published:** 2023-03-23

**Authors:** Manuel González-Cuesta, Alan Chuan-Ying Lai, Po-Yu Chi, I-Ling Hsu, Nien-Tzu Liu, Ko-Chien Wu, José M. García Fernández, Ya-Jen Chang, Carmen Ortiz Mellet

**Affiliations:** † Department of Organic Chemistry, Faculty of Chemistry, University of Seville, Seville E-41012, Spain; ‡ Institute of Biomedical Sciences, Academia Sinica, Nankang, Taipei 115, Taiwan; § Instituto de Investigaciones Químicas (IIQ), CSIC, Universidad de Sevilla, Américo Vespucio 49, Sevilla E-41092, Spain; ∥ Institute of Translational Medicine and New Drug Development, China Medical University, Taichung 404, Taiwan

## Abstract

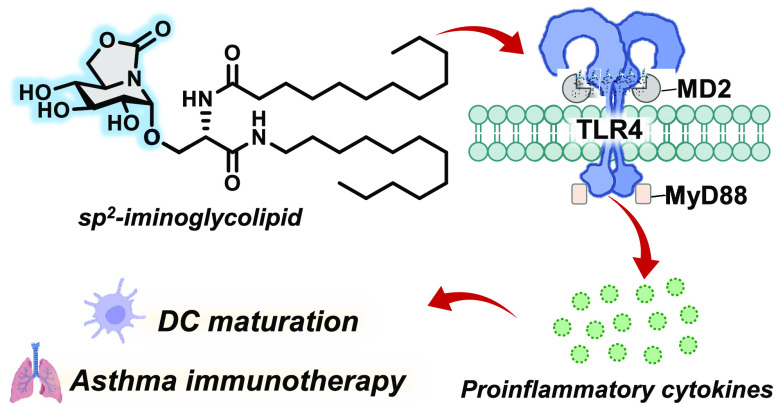

Glycolipids with TLR4 agonistic properties can serve either as therapeutic agents or as vaccine adjuvants by stimulating the development of proinflammatory responses. Translating them to the clinical setting is hampered by synthetic difficulties, the lack of stability in biological media, and/or a suboptimal profile of balanced immune mediator secretion. Here, we show that replacement of the sugar fragment by an sp^2^-iminosugar moiety in a prototypic TLR4 agonist, **CCL-34**, yields iminoglycolipid analogues that retain or improve their biological activity in vitro and in vivo and can be accessed through scalable protocols with total stereoselectivity. Their adjuvant potential is manifested in their ability to induce the secretion of proinflammatory cytokines, prime the maturation of dendritic cells, and promote the proliferation of CD8^+^ T cells, pertaining to a Th1-biased profile. Additionally, their therapeutic potential for the treatment of asthma, a Th2-dominated inflammatory pathology, has been confirmed in an ovalbumin-induced airway hyperreactivity mouse model.

## Introduction

Toll-like receptors (TLRs) are evolutionarily preserved type I transmembrane proteins that recognize conserved pathogen-associated molecular patterns (PAMPs) and danger-associated molecular patterns (DAMPs), representing the first line of host defense. The ability to sense molecular patterns makes TLRs vital regulators for innate immunity, further helping to shape the adaptive immune response by providing key signals to prime naïve CD4^+^ T cells toward a specific T helper (Th) profile, like cell-mediated response (Th1) or humoral immune response (Th2).^1−3^ Toll-like receptor 4 (TLR4) is one of the most important members of the whole TLR family. It is responsible for the detection of lipopolysaccharides (LPS) from Gram-negative bacteria and plays a key role in the nonspecific inflammatory reaction.

The role of TLR4 in driving Th1/Th2 immune response stimulation is complex and multifaceted. TLR4 activation can induce the release of both Th1 (e.g., interferon-γ; IFN-γ) and Th2 cytokines (e.g., interleukines-4 and 13; IL-4 and IL-13) in a context-dependent manner. The precise mechanisms at work are not fully understood, but it is believed to involve the activation of several downstream signaling pathways, including the myeloid differentiation primary response 88 (MyD88)-dependent and TIR-domain-containing adapter-inducing interferon-β (TRIF)-dependent pathways. The resulting Th1/Th2 polarization is further influenced by various factors, such as the type of TLR4 ligand, the duration and strength of TLR4 activation, and the presence of other co-stimulatory signals.^4,5^ Not surprisingly, dysregulated TLR4 signaling contributes to the pathogenesis of many chronic and acute inflammatory diseases, such as asthma, arthritis, cardiovascular disorders, cancer, and sepsis, underlining the importance of TLR4 as a target for therapeutic interventions.^6−8^ Both the agonistic and antagonistic aspects of TLR4 signaling pathways are being explored, and a number of clinical trials are currently in progress for a variety of diseases.^9^

**Figure 1:**
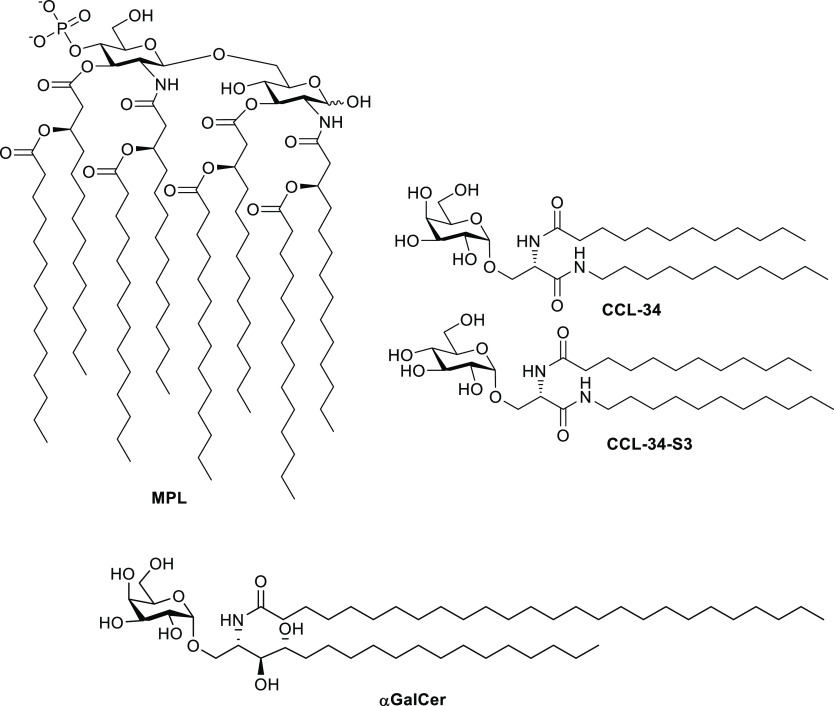
Structures of monophosphorylated glycolipid A (MPL), α-galactosylceramide (αGarlCer), and the serine-based glycolipids **CCL-34** and **CCL-34-S3**.

Efforts in discovering novel TLR4 agonists have mainly been directed toward the development of new vaccine adjuvants, with better properties, improved immune-stimulatory effects, and reduced toxicity, thanks to their ability to trigger a Th1-mediated immune response.^10,11^ The most prominent examples are anionic glycolipids related to lipid A, the TLR4 activating motif of bacterial LPS, isolated from either natural or synthetic sources.^12−16^ Despite extensive research in the field, monophosphoryl lipid A (MPL; Figure 1), a TLR4 agonist purified from *Salmonella minnesota* LPS, remains the only adjuvant used in licensed vaccines preventing human papillomavirus (HPV) and hepatitis B virus (HBV) infections.^17−19^ MPL is also the adjuvant component of Pollinex Quattro, an allergy vaccine containing the pollen extract which has entered phase II clinical trials for the monotherapy of allergic rhinitis and asthma.^20−23^ The underlying concept is that hematopoietic cells (HPCs) and nonhematopoietic stromal or structural airway cells (SACs) express TLR4 in qualitatively differentiated patterns: TLR4 signaling in SACs instructs dendritic cells (DCs) to induce Th2 responses, whereas on HPCs, this receptor can trigger a signaling pathway that programs DCs to polarize the immune response toward Th1 states. Consistent with this view, activation by TLR4 agonists could lead to the attenuation of allergen-triggered Th2 sensitization by enhancing the Th1 response, thus providing protection from airway hyperreactivity (AHR). Although improvement in nasal symptom scores was observed, a relatively high dose (100 μg) was required, and no inhibition of nasal allergen challenge responses compared with placebo was achieved; it was not pursued to phase III. It becomes apparent that exploiting the full potential of TLR4-associated immunity requires small-molecule TLR4 ligands with a specific function such as molecular probes to highlight the specific role of TLR4 in immune system regulation.

Serine-based glycolipids, of which **CCL-34** (Figure 1) is the archetype, have emerged as a new class of unnatural TLR4 agonists with a strong potential for drug development.^24^ Ironically, it was originally conceived as a structural analogue of α-galactosylceramide (α-GalCer or KRN7000; Figure 1), the prototypic ligand of the CD1d receptor in immune system cells. The corresponding CD1d−αGalCer complex binds the T-cell receptor (TCR) on invariant natural killer T cells (iNKT) to induce a strong immune response. **CCL-34** comprises a galactopyranose fragment α-linked to a serine-scaffolded hydrophobic lipid moiety that contains a linear C_12_ acyl substituent at the amine group and an *N*-undecyl amide chain at the carboxylic function of the amino acid component. Compared with other known TLR4 agonists, **CCL-34** has the advantages of a defined structure, easier synthesis, and less contamination with bioactive microbial components. It has been shown to modulate differentiation and maturation of myeloid DCs^25^ and to elicit anticancer immunity via TLR4.^26,27^ Structure–activity relationship studies demonstrated that the sugar portion plays an essential role in the biological properties. The configurational pattern of the monosaccharide admitted substantial variability. Thus, the glucosyl lipid derivative **CCL-34-S3** (Figure 1) exhibited a TLR4 agonist potency that was similar to that of **CCL-34**, suggesting that the stereochemistry of the 4-hydroxyl group is not crucial for activity. However, shifting from α- to β-anomeric configuration fully abolished the capability of activating TLR4-dependent signaling.^28^ The complications associated with the stereoselective synthesis of α-glycosides, generally requiring tedious and time-consuming procedures,^28−30^ and their instability to the action of α-glycosidases represent two important hurdles that may thwart the translation of these glycolipids to the clinic.

**Figure 2:**
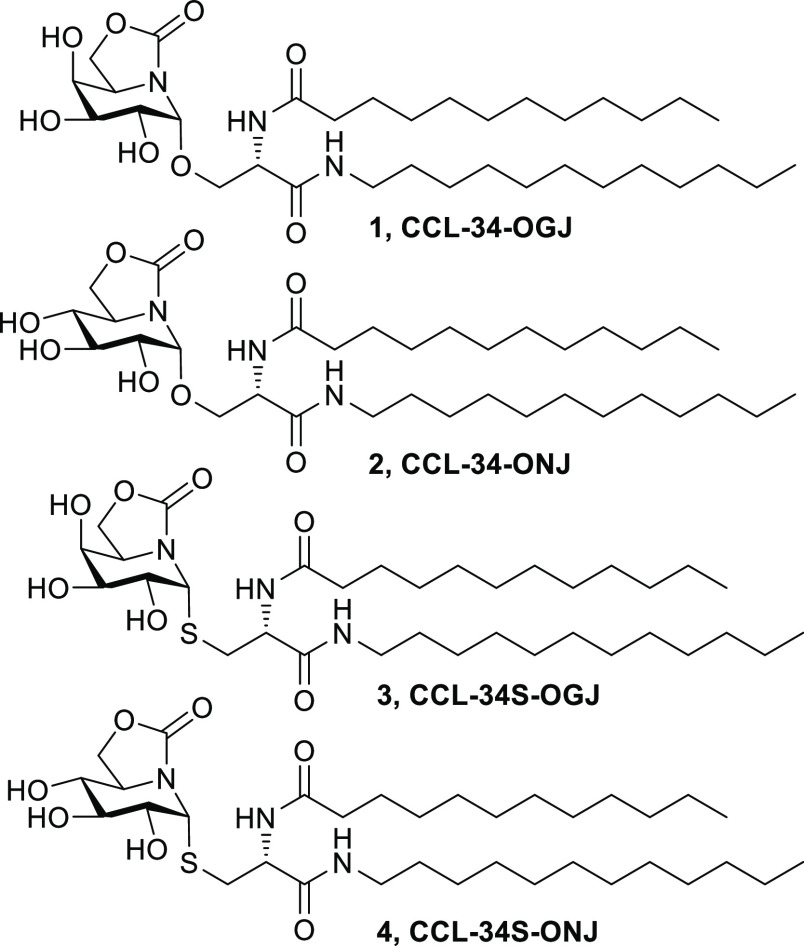
sp^2^-IGL structures **1–4** (**CCL-34-OGJ/ONJ** and **CCL-34S-OGJ/ONJ**).

We conceived that the abovementioned difficulties could be overcome by replacing the carbohydrate moiety in **CCL-34** with an sp^2^-iminosugar-type surrogate. sp^2^-Iminosugars are a unique family of glycomimetics characterized by the presence of a pseudoamide-type nitrogen (e.g., a carbamate, urea, thiocarbamate, thiourea, or guanidine nitrogen) in place of the endocyclic oxygen atom in monosaccharides. They emulate not only the structure and function but also the chemistry of monosaccharides, displaying an exacerbated anomeric effect.^31−34^ Notably, different from classical iminosugars, sp^2^-iminosugars can engage in *O* and *S*- glycosylation reactions, affording exclusively the corresponding α-*O*- or *S*-glycoside.^35,36^ This property has been previously exploited in the synthesis of specific glycosidase inhibitors and effectors,^37,38^ lectin ligands,^39−42^ mitogen-activated protein kinase (MAPK) regulators with anti-inflammatory,^43−46^ anticancer,^47−49^ and antiparasitic activities^50,51^ and tumor-associated carbohydrate antigen mimics,^52,53^ including a glycoconjugate-based anticancer vaccine.^54^ The structural resemblance of the diantennated lipid aglycone in **CCL-34** derivatives with the ceramide portion of endogenous glycolipid immunomodulators may enable additional mechanisms contributing to the resolution of the inflammatory process associated with allergy. Thus, glucosylceramide and glycosphingolipids are known to promote or inhibit TLR4-mediated signal transduction in a context-dependent manner by modifying the cell membrane properties.^55,56^ Notably, the corresponding α-anomers can further regulate the production of pro-and anti-inflammatory mediators by iNKT cells,^57^ a subset of innate-like T cells (CD1d-restricted T cells) that also contribute to allergic inflammation.^58,59^ With these considerations in mind, in the present study, we have synthesized serine- and cysteine-based sp^2^-iminoglycolipids (sp^2^-IGLs) featuring 5*N*,6*O*-oxomethylidene-galactonojirimycin (OGJ) and -nojirimycin (ONJ) glycone moieties, namely **CCL-34-OGJ** (**1**) and **CCL-34-ONJ** (**2**), or the *S*-glycoside analogues, **CCL-34S-OGJ** (**3**) and **CCL-34S-ONJ** (**4**), respectively (Figure 2). Assessment of their immunomodulatory properties showed that they represent a novel class of TLR4 agonists with the ability to counteract Th2 sensitization. Investigation of their potential in asthma immunotherapy in a murine model led to the identification of compound **2** as a promising candidate for drug development.

## Results and Discussion

### Synthesis of sp^2^-Iminoglycolipids

**Scheme 1:**
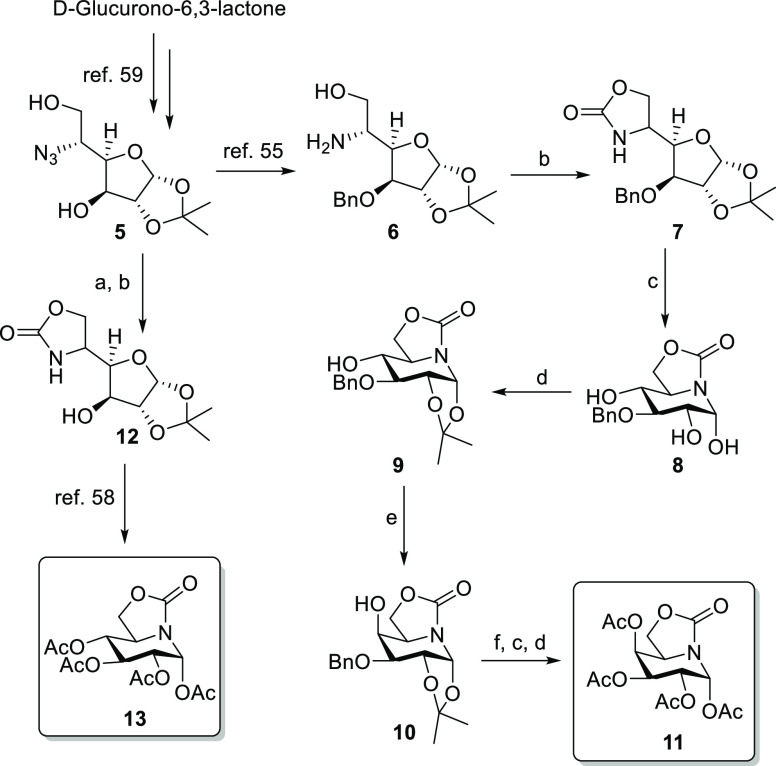
Synthesis of the sp^2^-Iminosugar Glycosyl Donors **11** and **13** Reagents and conditions: (a) PPh_3_ and THF–NH_4_OH, RT, 18 h, quant. (b) BTC, DCM, and DIPEA, RT, 1 h, 80%. (c) TFA–H_2_O 9:1, RT, 30 min, 75%. (d) PTSA and acetone, 4 Å MS, 50 °C, 4 h, 60%. (e) (i) Tf_2_O and Py, −30 °C, 1 h; (ii) NaNO_2_ and DCM, RT, 18 h, 50% (two steps). (f) H_2_, Pd/C, and MeOH, RT, 1 h, quant.

The preparation of the sp^2^-IGLs **1–4** implies the (thio)glycosylation of the corresponding serine or cysteine lipid with an appropriate OGJ or ONJ pseudoglycosyl donor. The corresponding per-*O*-acetates **11** and **13** were considered for this purpose. Previous syntheses of the parent sp^2^-iminosugars, limited to <100 mg batches, proved unsuitable for larger scale production, which seriously hampers access to the target conjugates in sufficient amounts to conduct the relevant in vitro and in vivo studies. Therefore, we first settled on an optimized route for the gram-scale preparation of **11** and **13** from commercial d-glucurolactone as a common starting material (Scheme 1).

For the synthesis of **11**, d-glucuronolactone was first converted into 5-amino-3-*O*-benzyl-5-deoxy-1,2-isopropylydene-α-d-glucofuranose **6** through known procedures (5 steps, 22% yield).^60^ Subsequent carbonylation with bis(trichloromethyl)carbonate and diisopropyl ethylamine afforded the bicyclic carbamate **7** (80% yield). Acid hydrolysis of the 1,2-*O*-acetal group in **7** with aqueous trifluoroacetic acid proceeded with concomitant furanose–piperidine rearrangement to give the bicyclic piperidine-carbamate compound **8**. Isopropylidenation of the 1,2-diol group (→**9**), followed by trifluoromethanesulfonylation of the 4-hydroxyl group with triflic anhydride and reaction of the crude triflate with sodium nitrite in dichloromethane, led to the galacto-configured OGJ derivative **10** in 50% yield.^61,62^ Removal of the benzyl and isopropylidene groups by sequential catalytic (Pd/C) hydrogenation and acid hydrolysis, followed by conventional acetylation, provided the OGJ peracetate **11** (5 steps from **6**, 18% overall yield, 2 grams of final compound per batch).

The gluco-configured glycosyl donor **13**, on his side, was prepared in eight steps on a 9 gram scale starting from 5-azido-5-deoxy-1,2-di-*O*-isopropylidene-α-d-glucofuranose **5**,^63^ readily accessible from commercial d-glucurono-6,3-lactone (4 steps, 64% yield).^64^ Reduction of the azide and carbonylation of the resulting vicinal aminodiol segment afforded the cyclic carbamate **12**. Hydrolysis of the acetonide group with aqueous TFA-promoted conversion gave the corresponding piperidine-carbamate sp^2^-iminosugar, which was acetylated to give the requested ONJ peracetate **13**.^65^ (Scheme 1).

**Scheme 2:**
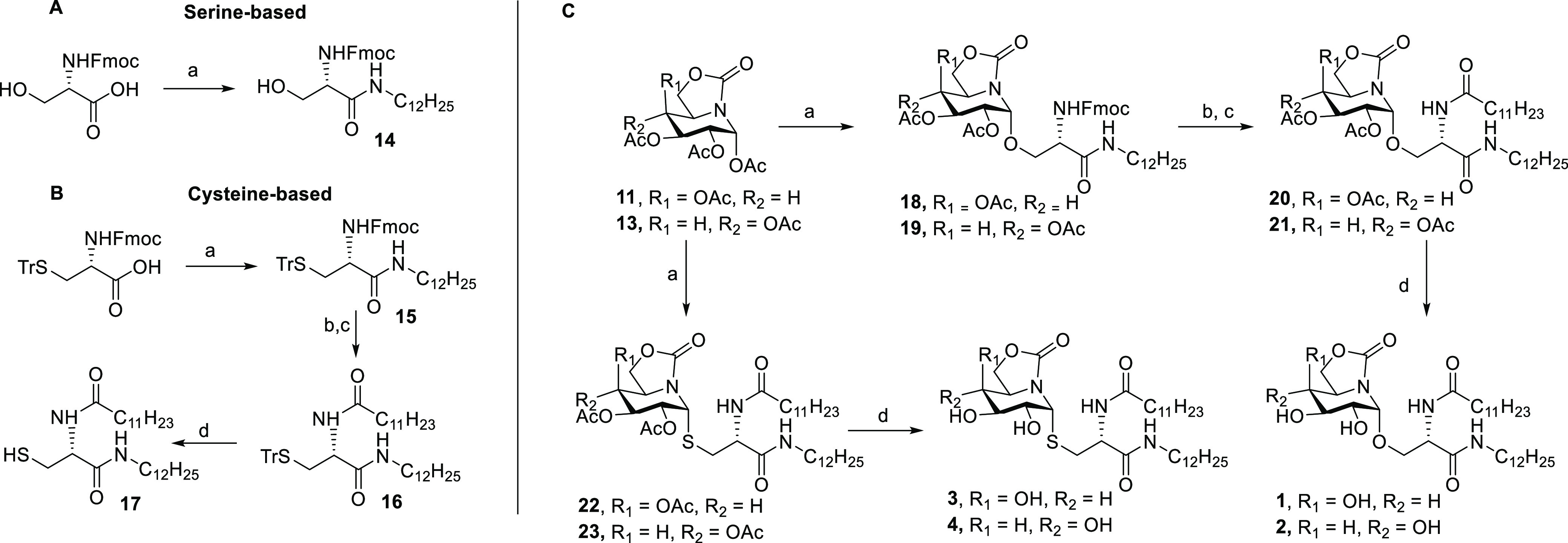
Synthesis of the sp^2^-IGL **1–4** Reagents and conditions: (A) (a) DCC, HOBt, dodecylamine, and THF, RT, 18 h, 90%. (B) (a) DCC, HOBt, dodecylamine, and THF, RT, 18 h, 90%. (b) Piperidine and THF, RT, 10 min. (c) Dodecanoyl chloride, Et_3_N, and DCM, RT, 18 h, 80% (two steps). (d) TFA–DCM and iPr_3_SiH, RT, 3 h, 90%. (C) (a) BF_3_·OEt_2_, **14** or **17**, and DCM, RT, 1 h, 63–65%. (b) Piperidine and THF, RT, 10 min. (c) Dodecanoyl chloride, Et_3_N, and DCM, RT, 18 h, 70–75% (two steps). (d) 1 M NaOMe and MeOH, RT, 1 h, quant.

The preparation of serine-derived lipid acceptors started with commercial *N*-Fmoc-protected serine (Fmoc–Ser–OH). An amidation reaction with dodecylamine, using DCC and HOBt as coupling reagents, provided the Fmoc-protected compound **14**. In the cysteine series, an analogous reaction sequence, starting in this case from the *N*-Fmoc-*S*-trityl-protected amino acid (Fmoc–Cys(Trt)–OH), afforded the corresponding dodecylamide **15** and the dianntenated intermediate **16**. The final treatment of **16** with TFA and triisopropylsilane (iPr_3_SiH), as a hydrogen source,^66^ yielded the glycosyl acceptor **17** (Scheme 2B)

The monoantennated serine lipid precursor **14** was engaged in the glycosidation of **11** and **13** using boron trifluoride etherate (BF_3_·OEt_2_) as the promoter. The corresponding *O*-glycoside derivatives **18** and **19** were thus obtained, with exclusive α-selectivity (1*R* configuration), in 63–65% yield. Removal of the Fmoc group under mild basic conditions, followed by amidation with lauric chloride and trimethylamine, furnished the **CCL-34** mimetics **20** and **21** in a 70–75% yield (two steps). Final deacetylation (NaOMe/MeOH) quantitatively provided **CCL-34-OGJ** and -**ONJ** sp^2^-IGL **1** and **2**, respectively.

The preparation of the cysteine analogues **3** and **4** was successfully achieved in a two-step sequence involving thioglycosidation of **11** or **13** with the diantennated cysteine lipid **17** in the presence of BF_3_·OEt_2_ (→**22** and **23**) followed by Zemplén deacetylation. Attempts to use the analogous diantennated serine lipid as an acceptor proved unpractical due to solubility issues. All the sp^2^-iminosugar derivatives (final compounds as well as intermediates) showed mass spectrometry and spectroscopic data (^1^H and ^13^C NMR) compatible with the proposed structures. Notably, the proton–proton-coupling constants about the six-membered piperidine ring supported that only the α-anomer was present, in agreement with the overwhelming anomeric effect in this family of compounds. The purity of the final compounds **1–4** has been confirmed to be >95% by RP–HPLC and combustion microanalysis.

### In Vitro Cytokine Signature, Adjuvancy Ability, and Signaling Route

Immunological evaluation of the sp^2^-IGLs **1–4** was performed first by assessing their ability to induce the secretion of proinflammatory cytokines in splenocytes isolated from B6 and myeloid differentiation primary response protein (MyD88) KO (MyD88^–/–^) mice. Since MyD88 is an intermediator of immune response signaling by all TLRs,^67^ depletion of inflammatory cytokine level expression on going from wild-type (WT) to MyD88^–/–^ splenocytes can be correlated with TLR involvement.

**Figure 3:**
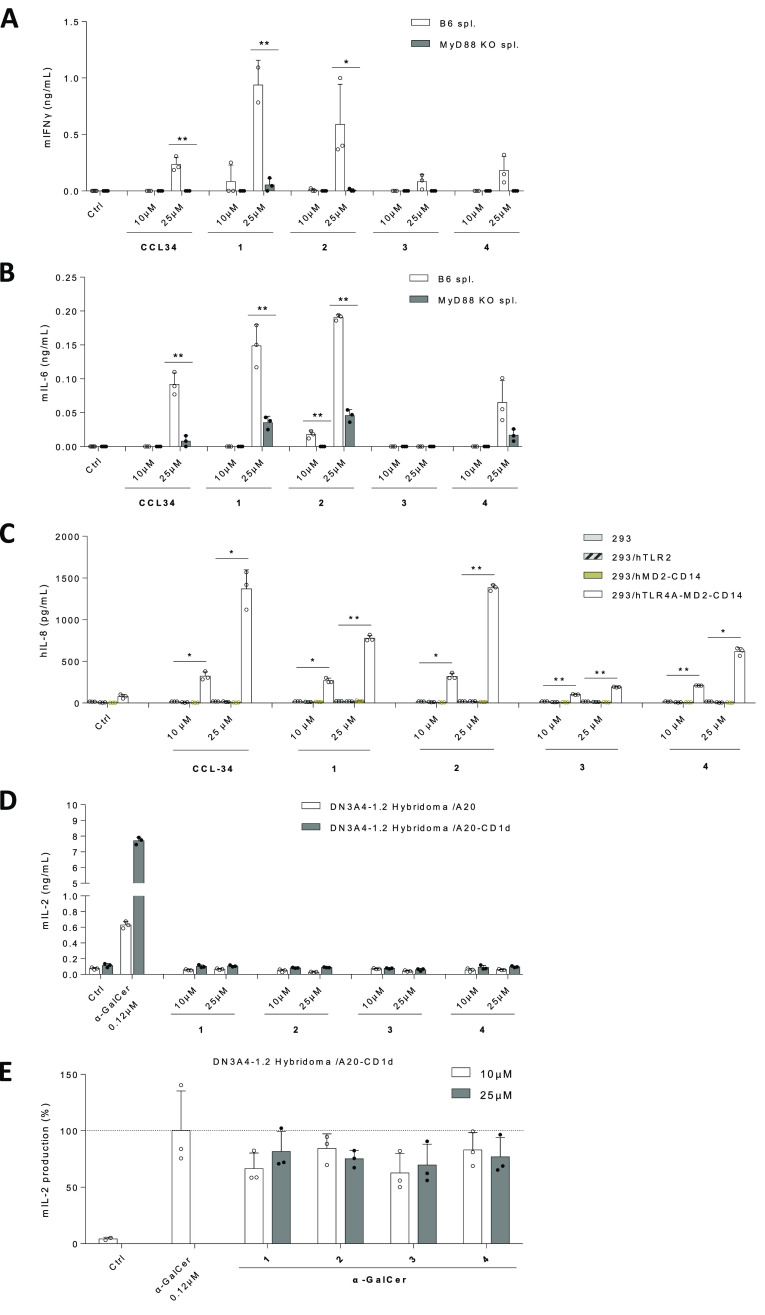
Immunostimulant function of sp^2^-IGL is TLR4-dependent but CD1d-independent. (A,B) Splenocytes isolated from B6 (C57BL/6JNarl) and MyD88 KO mice treated with compounds **1–4**, with IFNγ and IL-6 levels in the culture supernatant measured afterward. (C) HEK-293 (293), 293/hTLR2, 293/hMD2–CD14, and 293/hTLR4A–MD2–CD14 cells treated with compounds **1–4** to test for TLR4-dependent agonism, with human IL-8 detected only in the latter as an indicator of TLR4 signaling. (D) Mouse iNKT hybridoma DN3A4–1.2 cells cultured with A20 cells with CD1d expression to assess CD1d-dependent activation, and mouse IL-2 detected as an indicator of CD1d-dependent signaling. (E) DN3A4–1.2 cells co-cultured with A20–CD1d cells treated with our compounds upon addition to α-GalCer to test for the ability of compounds **1–4** to inhibit α-GalCer activity. Data are shown as means ± SEMs of two independent experiments (*n* = 3 each). *P < 0.05; **P < 0.01.

Binding of lipid A to the TLR4/MD-2 complex in immune cells triggers the production of inflammatory cytokines, such as IFNγ and IL-6.^68^ The serine-based glycolipid **CCL-34** likewise promotes an increase in the levels of these two cytokines in splenocytes.^24^ We found that the four sp^2^-IGL-type mimetics synthesized in this work displayed significantly stronger IFNγ expression than the parent compound used as the control in wild-type splenocytes from B6 mice (Figure 3A). The serine-based *O*-glycolipid mimetics **1** and **2** further outperformed **CCL-34** in enhancing IL-6 expression, whereas the cysteine-based *S*-glycolipid mimetics **3** and **4** were less efficient in this case (Figure 3B). We also found that MyD88^–/–^ mice splenocytes treated with the test compounds produced no or very low concentrations of IFNγ and IL-6, proving that their proinflammatory activity is MyD88-dependent (Figure 3A,B). TLR4-dependent signaling was next confirmed by using human embryonic kidney 293 (HEK-293; 293) cells engineered to express all components of the human TLR4 receptor complex (293/hTLR4A–MD-2–CD14), which is the key for signal transduction by ligands targeting TLR4 (Figure 3C). HEK-293 cells, which do not express any components of the TLR4 receptor complex (TLR4, MD-2, and CD14), were transfected with the genes for human TLR4, MD-2, and CD14. Nontransfected HEK-293 and HEK-293 expressing only the human MD2 and CD14 components (293/hMD-2–CD14) were used as controls. Upon treatment with compounds **1–4**, human IL-8 expression was detected in 293/hTLR4A–MD-2–CD14 cells, but not in the control cells, as an unequivocal indicator of TLR4 signaling. To verify the selectivity of TLR4 over TLR2 signaling, the compounds were also tested on HEK-293 cells expressing human TLR2 (293/hTLR2), and no agonist activity (no expression of the human IL-8 reporter molecule) was detected (Figure 3C). We also established that the treatment of compounds **1–4** did not cause a significant reduction in cell viability in B6 mouse splenocytes or HEK-293 cells and was well tolerated (MTT assay; Supporting Information Figure S1).

Given the structural resemblance of **CCL34** and the sp^2^-IGL-type mimetics **1–4** with αGalCer, discerning the off-target immune activation via the CD1d–TCR pathway is mandatory. By using the mouse iNKT hybridoma DN3A4–1.2 reporter system, we confirmed that none of the test compounds could activate this signaling route (no significant expression of the reporter interleukine IL-2 was detected; Figure 3D). Further, neither treatment with **CCL34** nor with any of the analogues **1–4** reduced IL-2 expression levels after α-GalCer stimulation of the mouse iNKT hybridoma, supporting that they do not compete with α-GalCer for CD1d binding (Figure 3E).

**Figure 4:**
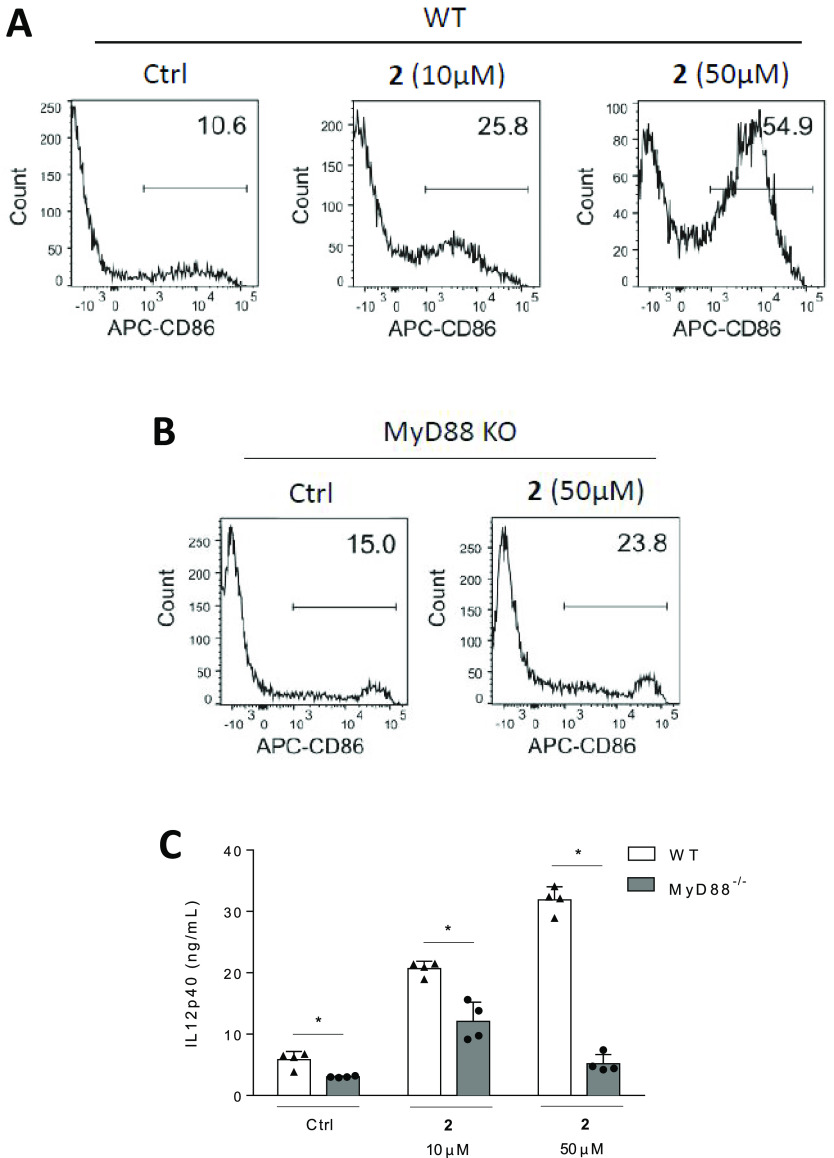
Compound **2**-promoted BMDC maturation is dose-dependent and MyD88-dependent. (A,B) BMDCs from B6 (WT) and MyD88 KO mice were treated with compound **2**, and the expression of CD86 was assessed using flow cytometry. (C) Expression of IL-12p40 in the culture supernatants of WT and MyD88 KO BMDCs was assessed. Data are shown as means ± SEMs of two independent experiments (*n* = 4 each). *P < 0.05.

The serine-based glycolipid mimetic **2**, exhibiting an immunostimulatory activity in vitro higher than the parent compound **CCL34**, was next assessed for its ability to induce the maturation of bone marrow-derived dendritic cells (BMDCs). Our results showed that compound **2** displayed a dose-dependent effect in inducing the expression of the cluster of differentiation 86 (CD86), a key mediator of T-cell activation and survival (Figure 4A) in WT mouse BMDCs.^69^ A parallel assay using BMDCs isolated from MyD88 KO mice evidenced much lower levels of CD86 upon treatment with 50 μM of **2**, consistent with a mechanism of action implying TLR4 (Figure 4B). Similarly, the levels of IL-12p40 secretion, a chemoattractant for macrophages, in the WT BMDC culture supernatant increased in the presence of compound **2** in a dose-dependent manner, which was not the case for MyD88^–/–^ BMDCs (Figure 4C).

**Figure 5:**
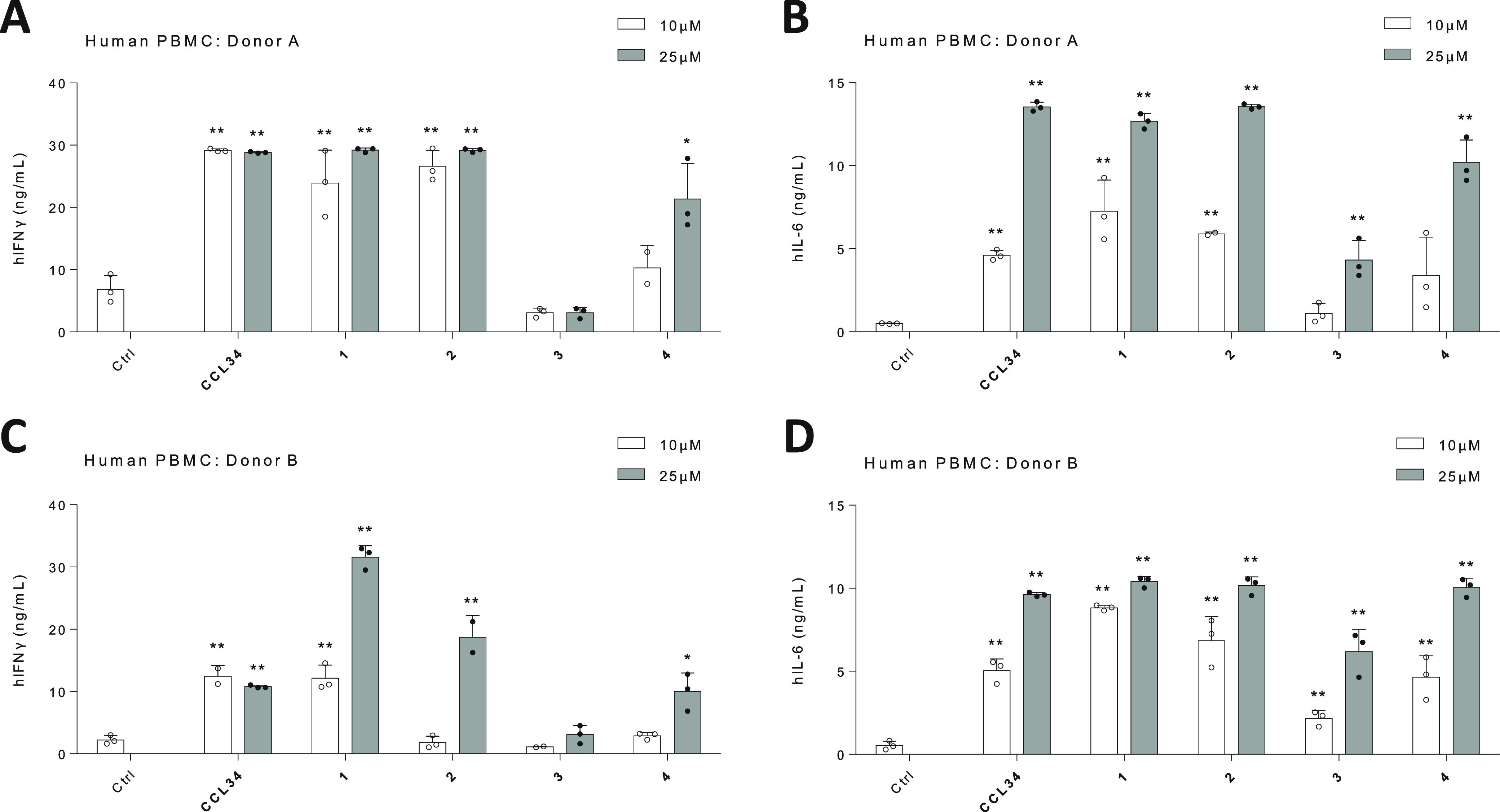
Compounds **1–4** induce strong IFNγ and IL6 expression in PMBCs from different donors. PBMCs from two donors were treated with compounds **1–4**. IFNγ in the culture supernatants of PBMCs from donors A (A) and B (C), and IL-6 in the culture supernatants from donors A (B) and B (D) were assessed. Data are shown as means ± SEMs of two independent experiments (*n* = 2–5 each). *P < 0.05; **P < 0.01.

The adjuvancy function of **CCL34** and compounds **1–4** was additionally evaluated in human peripheral blood mononuclear cells (PBMCs) from two different donors, A and B, via the detection of IFNγ and IL-6. Compounds **1** and **2** induced expression levels of both IFNγ (Figure 5A,C) and IL-6 (Figure 5B,D) that rivaled the performance of **CCL34**, supporting a T-helper 1 (Th1)-biased trait. The cysteine-based derivatives **3** and **4** exhibited lower immunostimulatory potency in this assay.

### In Vivo Evaluation of the Immunostimulatory Activity

**Figure 6:**
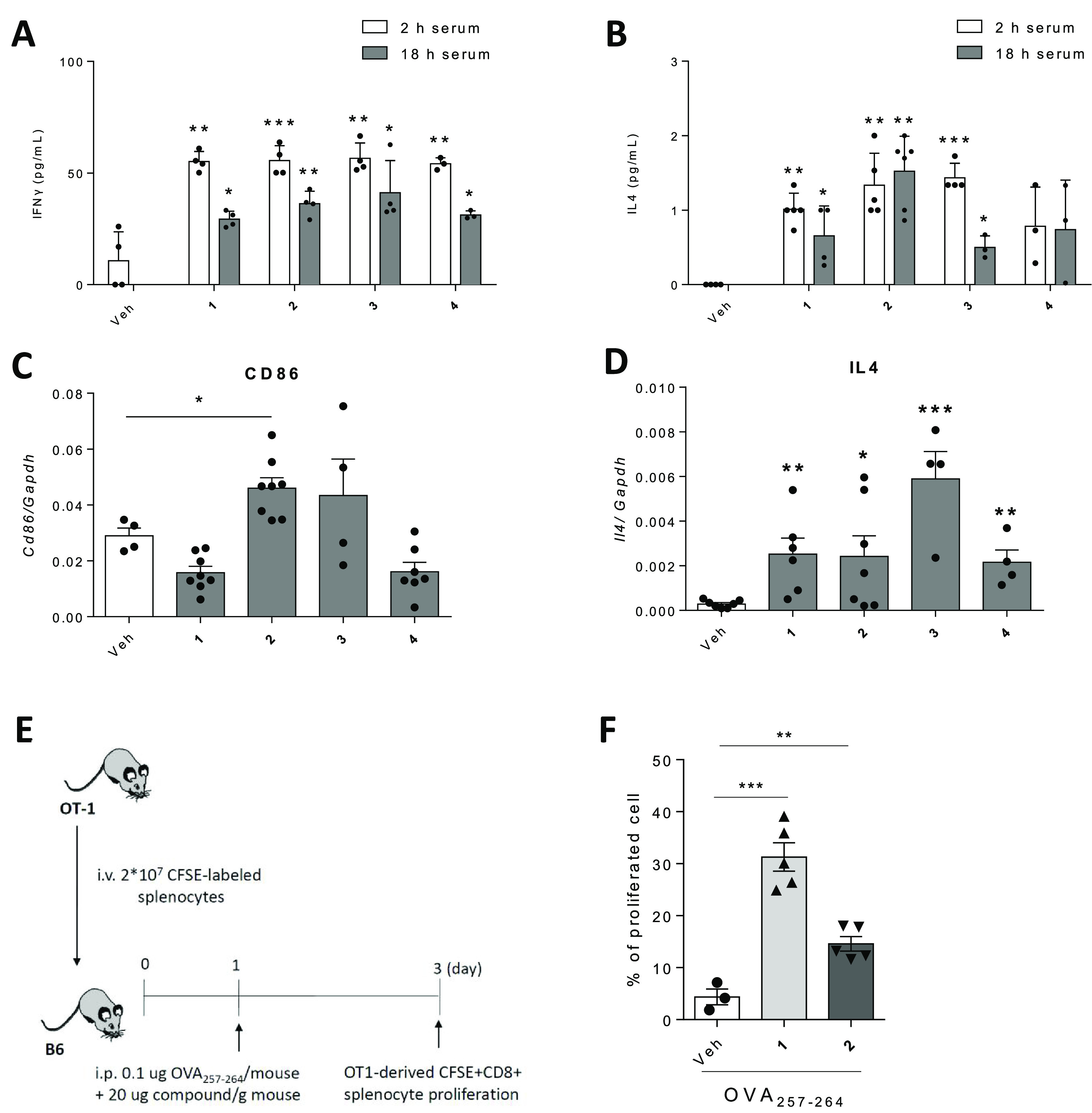
Compounds **1–4** possess strong adjuvancy function and promote DC and B cell maturation in WT mice and OVA-specific CD8^+^ T cell proliferation in the OT-1 adoptive transfer mouse model. (A–D) B6 mice treated with compounds **1–4** (20 μg compound/g mouse weight, i.p. administration). Sera were collected at 2 and 18 h post-treatment of compounds, and IFNγ (A) and IL-4 (B) protein concentrations were measured. Spleens from treated mice were also collected, and the mRNA levels of *Cd86* (C) and *Il4* (D), normalized to the glyceraldehyde-3-phosphate dehydrogenase (*Gaphd*) level, were analyzed. (E,F) Splenocytes isolated from OT-1 mice, in which OVA-specific CD8^+^ T cells could be expanded by the antigen OVA, labeled with CFSE dye, and adoptively transferred into B6 mice. Recipient B6 mice were then treated with OVA_257–264_ peptide mixed with compound **1** or **2**, and the proliferation of OVA-specific CD8^+^ T cells was analyzed using flow cytometry. (E) Experimental scheme. (F) Percentage of proliferating OVA-specific CD8^+^ T cells. Data are shown as means ± SEMs of two independent experiments (*n* = 3–8 each). *P < 0.05; **P < 0.01; ***P < 0.001.

The in vivo potential of the sp^2^-IGLs **1–4** as adjuvants was initially judged by determining their capacity to induce the production of IFNγ and IL-4 as archetypical mediators of humoral and immune responses, respectively, in WT mice.^70^ The levels of both cytokines in serum were significantly increased (Figure 6A,B) at 2 h post-treatment (20 μg compound/g mouse weight, intraperitoneal (i.p.) administration) and remained high after 18 h. Compound **2** was found to be the most efficient in the series at increasing the mRNA level of *Cd86* in the spleen, signifying its capability to promote DC maturation (Figure 6C). All compounds could also promote a rise in the mRNA level of *Il-4*, with compound **3** showing the strongest effect in this case (Figure 6D).

To demonstrate the adjuvant properties of the serine-based sp^2^-IGLs, we tested their ability to promote ovalbumin (OVA)-specific proliferation of CD8^+^ T cells in the OT-1 adoptive transfer mouse model.^71^ Briefly, splenocytes from this mouse model express an OVA-specific T cell receptor. Upon OVA binding, CD8^+^ T cell proliferation is induced, a characteristic feature of an adaptive immune response. We found that mixing OVA with either compound **1** or **2** significantly enhanced OVA-specific CD8^+^ T cell proliferation (Supporting Information Figures S2 and 6E) by 10- and 5-fold, respectively, supporting a strong immunostimulatory activity (Figure 6F).

### In Vivo Evaluation of the Immunotherapeutic Potential against Asthma

**Figure 7:**
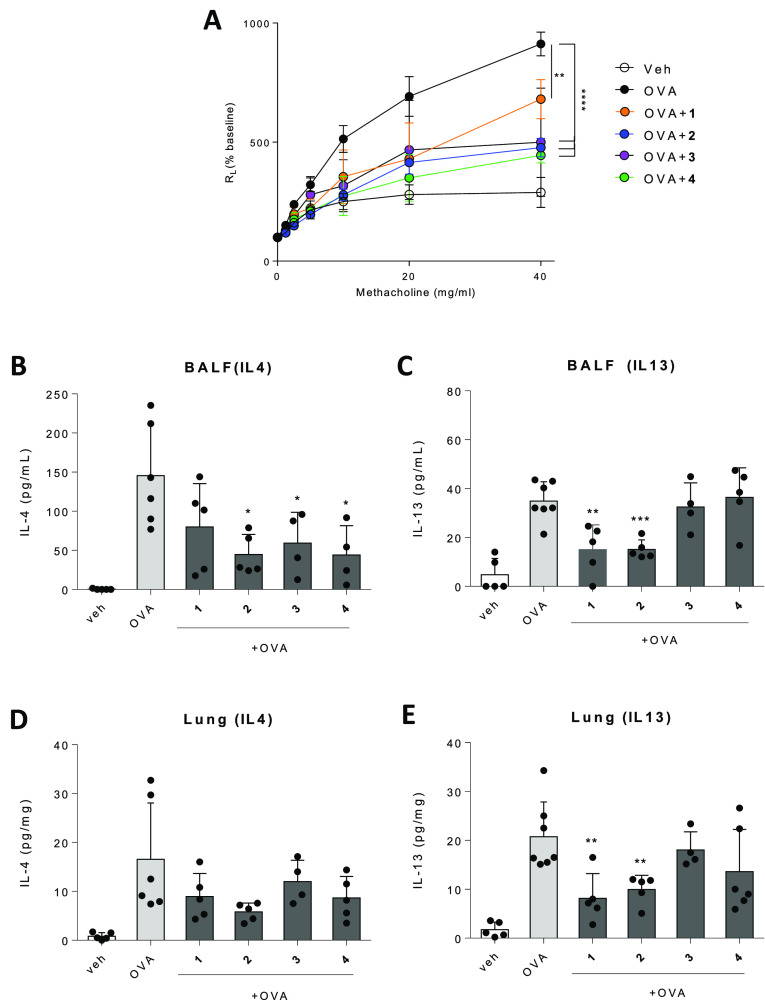
Treatment with compounds **1–4** attenuates OVA-induced AHR and airway inflammation. Compounds were used to treat BALB/c mice (20 μg/g mouse weight, i.p. administration), which were sensitized with OVA to induce AHR and airway inflammation. (A) Evaluation of AHR in OVA-sensitized mice treated with test compounds. (B,C) Levels of IL-4 (B) and IL-13 (C) in BALF. (D,E) Levels of IL-4 (D) and IL-13 (E) in BALF. Data are shown as means ± SEMs of two independent experiments (*n* = 5–7 each). *P < 0.05; **P < 0.01; ***P < 0.001.

The ability of compounds **1–4** to induce high expression levels of IFNγ in vivo through the TLR4 pathway, meaning a Th1-biased profile, suggests their potential to counteract Th2-dominated inflammatory pathologies, such as asthma.^72^ We have explored this notion in the OVA-induced AHR mouse model.^73^ After OVA sensitization, compounds **1–4** (20 μg/g mouse weight, i.p.) were used to treat mice. With the administration of increasing concentrations of methacoline, these mice developed bronchoconstriction and consequently enhanced pulmonary resistance (*R*_L_). Interestingly, *R*_L_ values were curtailed by as much as 50% upon treatment with compounds **1–4** (Figure 7A). Additionally, determination of the levels of the Th2 cytokines IL-4 and IL-13, which are considered instigators of AHR and airway inflammation, in the bronchoalveolar fluid (BALF) and in the lungs was additionally conducted. We found that all compounds reduced the expression levels of IL-4 in BALF, although data only reached significance for compounds **2–4** (Figure 7B), and a similar trend was observed in the lungs. In the case of IL-13, only the serine-based derivatives **1** and **2** were efficient at decreasing expression levels in both BALF and lungs, which agrees with their stronger Th1-biased immunomodulatory character (Figure 7C).

## Conclusions

The results above-discussed show that (thio)glycosidation of sp^2^-iminosugar glycosyl donors with serine- or cysteine-based lipid acceptors provides an efficient entry to bioactive glycolipid mimetics, namely sp^2^-IGLs. In these compounds, the sugar portion is replaced by a piperidine–carbamate bicyclic moiety, whose hydroxylation and configurational profiles can be predetermined. Notably, the glycosylation reaction proceeds with total α-stereoselectivity, which was here exploited to synthesize sp^2^-IGL analogues of the serine-based glycolipid **CCL-34**, a non-LPS-related TLR4 agonist. Evaluation of the immunomodulatory properties of the new sp^2^-IGLs supports their TLR4 agonistic character both in vitro and in vivo, with a cytokine signature characterized by high expression levels of IFNγ, suggesting a Th1-biased profile. Interestingly, the compounds were found to counteract the Th2 proinflammatory context in a mouse model of OVA-induced respiratory hyperreactivity mimicking asthma, promoting crisis resolution. They possess well-defined chemical structures, are chemically stable, are expected to be metabolically inert, and can be prepared through efficient and scalable experimental procedures suitable for optimization strategies. Investigation of this novel family of immunomodulators in other immune-compromised pathologies, such as infectious diseases and cancer, is currently sought in our laboratories.

## Experimental Section

### Chemistry

All chemicals were of reagent grade and used without further purification unless otherwise specified. The experimental data for 5-azido-5-deoxy-1,2-*O*-isopropylidene-α-d-glucofuranose (**5**),^60^ 5-amino-5-deoxy-1,2-*O*-isopropylidene-α-d-glucofuranose 5,6-(cyclic carbamate) (**12**),^65^ (1*R*)-1,2,3,4-tetra-*O*-acetyl-5*N*,6*O*-oxomethylidenegalactonojirimycin (**11**),^74^ and (1*R*)-1,2,3,4-tetra-*O*-acetyl-5*N*,6*O*-oxomethylidenenojirimycin (**13**)^65^ were identical to those previously reported. **CCL34** was prepared as previously described.^28^ Optical rotations were measured at 20 ± 2 °C in 1 dm tubes on a Jasco P-2000 polarimeter using a sodium lamp (λ 589 nm). IR spectra were recorded on a JASCO FTIR-410 device. UV spectra were recorded on a JASCO V-630 instrument; the unit for ε values: mM^–1^ cm^–1^. ^1^H (and ^13^C NMR) spectra were recorded at 500 (125.7) MHz with a Bruker 500 DRX magnet. 1D TOCSY, 2D COSY, HMQC, and HSQC experiments were used to assist with NMR assignments. Thin-layer chromatography (TLC) was carried out on aluminum sheets coated with silica gel 60 F_254_ Merck with visualization by UV light and by charring with ethanolic 10% H_2_SO_4_ and 0.1% ninhydrin. Column chromatography was carried out on Silice 60 A.C.C. Chromagel (SDS 70–200 and 35–70 μm) for preparative purposes. Mass spectra were carried out on a Bruker Daltonics Esquire6000. The samples were introduced via a solid probe heated from 30 to 280 °C. ESI was used as the ionization source (electrospray ionization), and methanol was used as the solvent. The samples were introduced via direct injection using a Cole-Parmer syringe at a flow rate of 2 μL/min. Ions were scanned between 300 and 3000 Da with a scan speed of 13,000 Da/s at unit resolution using resonance ejection at the multipole resonance of one-third of the radio frequency (Ω = 781.25 kHz). High-resolution mass spectrometer (HRMS) spectra were recorded on a Bruker MaXis Impact or Agilent Technologies 6210 time-of-flight LC/MS spectrometers using positive or negative ESI. Elemental analyses were carried out at the Instituto de Investigaciones Químicas (Sevilla, Spain) using an elemental analyser, the Leco CHNS-932. The analytical results for C, H, N, and S were within ±0.4 of the theoretical values.

#### *N*α-Lauryl-*O*-(1*R*-5*N*,6*O*-oxomethylidenegalactonojirimycinyl)-*N*-dodecyl-l-serinamide (**1**)

Compound **1** was obtained by the treatment of a solution of **20** (45 mg, 0.058 mmol) in MeOH (1 mL) with methanolic 1 M NaOMe (42 μL), followed by neutralization with the Amberlite IR120 H^+^ cation-exchange resin, filtration, and evaporation. Yield: 33 mg (quant). *R*_f_ 0.60 (70:10:1 DCM–MeOH–H_2_O). [α]_D_ + 15.6 (*c* 1.0 in 5:1 DCM–MeOH). ^1^H NMR (500 MHz, 1:1 MeOD–CDCl_3_): δ 5.13 (d, 1H, *J*_1_,_2_ = 4.2 Hz, H-1), 4.55 (dd, 1H, *J*_4,5_ = 7.5 Hz, *J*_4,5_ = 5.9 Hz, H-4), 4.43 (m, 1H, OCH_2_CH), 3.97 (m, 2H, OCH_2_CH), 3.82 (m, 2H, H-2, H-6a), 3.73 (m, 2H, H-3, H-6b), 3.65 (m, 1H, H-5), 3.19 (m, 2H, NHCH_2_), 2.24 (m, 2H, COCH_2_), 1.87–0.88 (m, 48H, CH_2_, CH_2_CH_3_). ^13^C NMR (125.5 MHz, 1:1 CD_3_OD–CDCl_3_): δ 174.6, 170.3 (CO_amide_), 157.7 (CO_carbamate_), 82.5 (C-1), 70.0 (C-2), 68.7 (C-3), 67.8 (C-6), 67.7(C-5), 63.7 (OCH_2_CH), 52.8 (C-4), 52.6 (OCH_2_CH), 39.5 (NHCH_2_), 35.8 (COCH_2_), 31.7–13.5 (CH_2_, CH_2_CH_3_). ESIMS *m*/*z*: 664.45 [M + Na]^+^. HRMS (ESI) calcd for [C_34_H_63_O_8_N_3_Na]^+^, 664.4507; found, 664.4503.

#### *N*α-Lauryl-*O*-(1*R*-5*N*,6*O*-oxomethylidenenojirimycinyl)-*N*-dodecyl-l-serinamide (**2**)

Compound **2** was obtained by the treatment of a solution of **21** (40 mg, 0.052 mmol) in MeOH (1 mL) with 1 M NaOMe in MeOH (42 μL), followed by neutralization with the Amberlite IR120 H^+^ cation-exchange resin, filtration, and evaporation. Yield: 30 mg (quant). *R*_f_ 0.60 (70:10:1 DCM–MeOH–H_2_O). [α]_D_ + 14.5 (*c* 1.0 in 5:1 DCM–MeOH). ^1^H NMR (500 MHz, 1:1 MeOD–CDCl_3_): δ 5.00 (d, 1H, *J*_1_,_2_ = 4.0 Hz, H-1), 4.44 (m, 1H, H-6a), 4.15 (dd, 1H, *J*_5_,_6b_ = 9.5 Hz, *J*_6a_,_6b_ = 6.3 Hz, H-6b), 3.64 (m, 1H, H-5), 3.59 (m, 3H, H-4, OCH_2_CH), 3.49 (bt, 1H, *J*_2,3_ = 10.7 Hz, H-3), 3.36 (dd, 1H, *J*_2,3_ = 9.9 Hz, H-2), 3.25 (m, 1H, OCH_2_CH), 3.11 (m, 2H, NHCH_2_), 2.15 (t, 2H, *J*_H,H_ = 7.0 Hz, COCH_2_), 1.53–0.79 (m, 48H, CH_2_, CH_2_CH_3_). ^13^C NMR (125.7 MHz, 1:1 CD_3_OD–CDCl_3_): δ 174.9, 170.5 (CO ester), 157.4 (CO_carbamate_), 82.7 (C-1), 74.4 (C-3), 73.5 (OCH_2_CH), 71.9 (C-2), 68.5 (C-5), 67.5 (C-6), 53.6 (C-4), 52.7 (OCH_2_CH), 39.9 (NHCH_2_), 36.3 (COCH_2_), 31.9–14.1 (CH_2_, CH_2_CH_3_). ESIMS *m*/*z*: 664.45 [M + Na]^+^. HRMS (ESI) calcd for [C_34_H_63_O_8_N_3_Na]^+^, 664.4507; found, 664.4511, Anal. Calcd for C_34_H_63_N_3_O_8_: C, 63.62; H, 9.89; N, 6.55. Found: C, 63.41; H, 9.74; N, 6.29.

#### *N*α-Lauryl-*O*-(1*R*-5*N*,6*O*-oxomethylidenegalactonojirimycinyl)-*N*-dodecyl-l-cysteinamide (**3**)

Compound **3** was obtained by the treatment of a solution of **22** in MeOH (2.6 mL) with 1 M NaOMe in MeOH (42 μL), followed by neutralization with the Amberlite IR120 H^+^ cation-exchange resin, filtration, evaporation, and freeze drying. Yield: 22 mg (quant). *R*_f_ 0.61 (70:10:1 DCM–MeOH–H_2_O). [α]_D_ + 20.12 (*c* 1.0 in DCM/MeOH). ^1^H NMR (500 MHz, 5:1 CD_3_OD–CDCl_3_): δ 5.30 (d, 1H, *J*_1_,_2_ = 5.8 Hz, H-1), 4.39 (m, 3H, H-6a, H-4, H-2), 4.08 (m, 1H, CH), 3.98 (m, 1H, *J*_6a,6b_ = 9.6 Hz, *J*_5,6b_ = 5.7 Hz, H-6b), 3.75 (bt, 1H, H-3), 3.54 (dd, 1H, *J*_5,6_ = 10.0 Hz, *J*_4,5_ = 2.6 Hz H-5), 3.09 (m, 2H, NHCH_2_), 2.84, 2.75 (dd, 2H, SCH_2_), 2.16 (t, 2H, COCH_2_), 1.53–0.79 (m, 48H, CH_2_, CH_2_CH_3_). ^13^C NMR (125.7 MHz, 5:1 CD_3_OD–CDCl_3_): δ 174.7, 170.7 (CO), 157.8 (CO_carbamate_), 70.7 (C-1), 68.4 (C-2), 67.1 (C-3), 63.6 (C-5), 62.8 (C-6), 53.2 (CH), 52.7 (C-4), 39.4 (NHCH_2_), 35.9 (COCH_2_), 31.7–13.5 (CH_2_, CH_2_CH_3_). ESIMS *m*/*z*: 658.34 [M + H]^+^. Anal. Calcd for C_34_H_63_N_3_O_7_S: C, 62.07; H, 9.65; N, 6.39; S, 4.87. Found: C, 61.88; H, 9.61; N, 6.19; S, 4.65.

#### *N*α-Lauryl-*O*-((1*R*)-5*N*,6*O*-oxomethylidenenojirimycinyl)-*N*-dodecyl-l-cysteinamide (**4**)

Compound **4** was obtained by the treatment of a solution of **23** in MeOH (2.6 mL) with 1 M NaOMe in MeOH (42 μL), followed by neutralization with the Amberlite IR120 H^+^ cation-exchange resin, filtration, evaporation, and freeze drying. Yield: 20 mg (quant). *R*_f_ 0.50 (70:10:1 DCM–MeOH–H_2_O). [α]_D_ + 16.23 (*c* 1.0 in DCM/MeOH). ^1^H NMR (500 MHz, 5:1 CD_3_OD–CDCl_3_): δ 5.20 (d, 1H, *J*_1,2_ = 5.7 Hz, H-1), 4.51 (bt, 1H, *J*_5,6a_ = 9.5 Hz, *J*_6a,6b_ = 8.6 Hz, H-6a), 4.40 (dd, 1H, *J*_5,6b_ = 6.3 Hz, H-6b), 4.18 (m, 1H, H-5), 3.79 (m, 1H, CH), 3.58 (dd, 1H, *J*_3,4_ = 9.5 Hz, *J*_4,5_ = 5.5 Hz, H-4), 3.40 (t, 1H, *J*_2,3_ = 9.3 Hz, H-3), 3.22 (m, 1H, H-2), 3.09 (m, 2H, NHCH_2_), 2.86, 2.78 (dd, 2H, SCH_2_), 2.16 (t, 2H, *J*_H,H_ = 7.0 Hz, COCH_2_), 1.53–0.79 (m, 48H, CH_2_, CH_2_CH_3_). ^13^C NMR (125.7 MHz, 4:1 CD_3_OD–CDCl_3_): δ 174.7, 170.7 (CO), 157.1 (CO_carbamate_), 74.1 (C-1), 73.8 (C-3), 71.1 (C-2), 67.1 (C-5), 62.6 (C-6), 53.3 (C-4), 53.2 (CH), 39.3 (NHCH_2_), 35.8 (COCH_2_), 32.7–13.3 (CH_2_, CH_2_CH_3_). ESIMS *m*/*z*: 658.33 [M + H]^+^. Anal. Calcd for C_13_H_16_N_4_O_8_: C, 43.82; H, 4.53; N, 15.73. Found: C, 43.89; H, 4.60; N, 15.57.

#### 5-Amino-5-deoxy-1,2-*O*-isopropylidene-3-*O*-benzyl-α-d-glucofuranose (**6**)

To a stirred solution of **5** (5.7 g, 17.0 mmol) in 5:1 dioxane–MeOH (230 mL), Ph_3_P (8.9 g, 34.0 mmol) was added, and the reaction mixture was stirred at room temperature for 4 h. NH_4_OH (32 mL) was added, and the mixture was stirred overnight. Then, the solvent was removed under reduced pressure, and the residue was purified by column chromatography (EtOAc, 45:5:1 → 45:5:3 EtOAc–EtOH–H_2_O). Yield: 7.1 g (90%). *R*_f_ 0.41 (45:5:3 EtOAc–EtOH–H_2_O). [α]_D_ – 68.2 (*c* 0.8 in DCM). ^1^H NMR (400 MHz, CDCl_3_): δ 7.45–7.30 (m, 5H, CH_arom_), 5.94 (d, 1H, *J*_1,2_ = 3.8 Hz, H-1), 4.76 (d, 1H, ^2^*J*_H,H_ = 11.8 Hz, OCH_2_Ph), 4.64 (d, 1H, H-2), 4.51 (d, 1H, OCH_2_Ph), 4.03 (d, 1H, *J*_3,4_ = 3.0 Hz, H-3), 3.99 (dd, 1H, *J*_4,5_ = 8.4 Hz, H-4), 3.79 (dd, 1H, *J*_6a,6b_ = 10.8 Hz, *J*_5,6a_ = 4.0 Hz, H-6a), 3.58 (dd, 1H, *J*_5,6b_ = 6.0 Hz, H-6b), 3.29 (m, 1H, H-5), 2.00 (bs, 1H, OH), 1.50, 1.34 (2 s, 6H, CMe_2_). ^13^C NMR (100 MHz, CDCl_3_): δ 137.2–128.0 (C_arom_), 111.6 (CMe_2_), 105.1 (C-1), 81.7 (C-2), 81.6 (C-3, C-4), 71.7 (OCH_2_Ph), 64.3 (C-6), 50.9 (C-5), 26.7, 26.2 (CMe_2_). ESIMS *m*/*z*: 310.1 [M + H]^+^. Anal. Calcd for C_16_H_23_NO_5_: C, 62.12; H, 7.49; N, 4.53. Found: C, 61.88; H, 7.27; N, 4.44.

#### 5-Amino-5-deoxy-1,2-*O*-isopropylidene-3-*O*-benzyl-α-d-glucofuranose 5,6-(Cyclic carbamate) (**7**)

To a stirred solution of amine **6** (7.3 g, 23 mmol) in DCM (162 mL), DIPEA (39 mL, 236 mmol) and triphosgene (10.5 g, 35.4 mmol) were added at 0 °C. After 30 min, the solvent was removed under reduced pressure, and the residue was purified by column chromatography using EtOAc as the eluent. Yield: 5.4 g (69%). *R*_f_ 0.84 (EtOAc). [α]_D_ – 91.7 (*c* 0.96 in CHCl_3_). ^1^H NMR (400 MHz, CDCl_3_): δ 7.50–7.30 (m, 5H, CH_arom_), 5.95 (d, 1H, *J*_1,2_ = 3.8 Hz, H-1), 5.38 (bs, 1H, NH), 4.74 (d, 1H, ^2^*J*_H,H_ = 11.8 Hz, OCH_2_Ph), 4.68 (d, 1H, H-2), 4.51 (d, 1H, OCH_2_Ph), 4.51–4.46 (m, 1H, H-6a), 4.43 (dd, 1H, *J*_6b,6a_ = 8.8 Hz, *J*_5,6b_ = 4.8 Hz, H-6b), 4.20 (dd, 1H, *J*_4,5_ = 7.8 Hz, *J*_3,4_ = 3.4 Hz, H-4), 4.12–4.05 (m, 1H, H-5), 4.02 (d, 1H, H-3), 1.52, 1.35 (2 s, 6H, CMe_2_). ^13^C NMR (100 MHz, CDCl_3_): δ 160.0 (CO), 137.1–128.0 (C_arom_), 112.2 (CMe_2_), 105.5 (C-1), 82.0 (C-2), 81.6 (C-4), 81.3 (C-3), 71.8 (OCH_2_Ph), 68.1 (C-6), 50.8 (C-5), 26.9, 26.3 (CMe_2_). ESIMS *m*/*z*: 358.1 [M + Na]^+^. Anal. Calcd for C_17_H_21_NO_6_: C, 60.89; H, 6.31; N, 4.18. Found: C, 60.93; H, 6.24; N, 4.09.

#### (1*R*)-3-*O*-Benzyl-1,2,3-trihydroxy-5*N*,6*O*-oxomethylidenenojirimycin (**8**)

A solution of **7** (2.1 g, 6.2 mmol) in 90% TFA–H_2_O (9.8 mL) was stirred at room temperature for 30 min. The solvent was eliminated under reduced pressure, co-evaporated several times with water, treated with NaOH 0.1 N until pH 8, and concentrated. The resulting residue was purified by column chromatography using EtOAc as the eluent, concentrated, and freeze-dried. Yield: 1.6 g (90%). *R*_f_ 0.60 (EtOAc). [α]_D_ – 2.7 (*c* 1.0 in H_2_O). ^1^H NMR (400 MHz, D_2_O): δ 7.50–7.30 (m, 5H, CH-arom), 5.33 (d, 1H, *J*_1,2_ = 4.0 Hz, H-1), 4.84 (d, 1H, ^2^*J*_H,H_ = 10.8 Hz, OCH_2_Ph), 4.80 (d, 1H, OCH_2_Ph), 4.57 (t, 1H, *J*_6a,6b_ = *J*_5,6a_ = 8.8 Hz, H-6a), 4.25 (dd, 1H, *J*_5,6b_ = 6.8 Hz, H-6b), 3.98–3.90 (m, 1H, H-5), 3.70 (t, 1H, *J*_2,3_ = *J*_3,4_ = 9.4 Hz, H-3), 3.63 (dd, 1H, H-2), 3.56 (t, 1H, *J*_4,5_ = 9.4 Hz, H-4). ^13^C NMR (100 MHz, D_2_O): δ 157.6 (CO), 137.6–128.4 (C_arom_), 80.9 (C-3), 75.3 (OCH_2_Ph), 74.6 (C-1), 73.3 (C-4), 71.1 (C-2), 67.6 (C-6), 53.2 (C-5). ESIMS *m*/*z*: 318.1 [M + Na]^+^. Anal. Calcd for C_14_H_17_NO_6_: C, 56.94; H, 5.80; N, 4.74. Found: C, 56.75; H, 5.63; N 4.57.

#### (1*R*)-3-*O*-Benzyl-4-hydroxy-1,2-*O*-isopropylidene-5*N*,6*O*-oxomethylidenenojirimycin (**9**)

A mixture of **8** (277 mg, 0.94 mmol), acetone (40 mL), and toluene-*p*-sulfonic acid monohydrate (205 mg, 1.08 mmol) was stirred at 50 °C for 1 h. The reaction mixture was diluted with EtOAc (20 mL) and washed with aq NaHCO_3_ (10 mL). The organic layer was dried (MgSO_4_), filtered, and concentrated. The resulting residue was purified by column chromatography (4:1 EtOAc–cyclohexane). Yield: 210 mg (67%). *R*_f_ 0.67 (9:1 EtOAc–cyclohexane). [α]_D_ + 4.3 (*c* 1.0 in DCM). ^1^H NMR (300 MHz, CDCl_3_): δ 7.50–7.30 (m, 5 H, CH-arom), 5.83 (d, 1H, *J*_5,6_ = 6.0 Hz, H-1), 5.00 (d, 1H, ^2^*J*_H,H_ = 12.0 Hz, OCH_2_Ph), 4.66 (d, 1H, OCH_2_Ph), 4.53 (t, 1H, *J*_6a,6b_ = *J*_5,6a_ = 9.0 Hz, H-6a), 4.31 (dd, 1H, *J*_5,6b_ = 5.5 Hz, H-6b), 4.23 (t, 1H, *J*_2,3_ = 6.0 Hz, H-2), 3.89 (m, 1H, H-5), 3.56 (dd, 1H, *J*_3,4_ = 9.0 Hz, H-3), 3.44 (t, 1H, H-4), 1.57 (s, 3H, CH_3_), 1.47 (s, 3H, CH_3_). ^13^C NMR (75.5 MHz, CDCl_3_): δ 156.1 (CO), 137.6–128.1 (C_arom_), 109.4 (CMe_2_), 82.6 (C-3), 80.5 (C-1), 76.4 (C-2), 73.2 (OCH_2_Ph), 71.3 (C-4), 66.2 (C-6), 53.0 (C-5), 27.9, 26.7 (CMe_2_). ESIMS *m*/*z*: 358.2 [M + Na]^+^. Anal. Calcd for C_17_H_21_NO_6_: C, 60.89; H, 6.31; N, 4.18. Found: C, 60.67; H, 6.18; N, 3.97.

#### (1*R*)-3-*O*-Benzyl-4-hydroxy-1-*O*-isopropylidene-5*N*,6*O*-oxomethylidene-galactonojirimycin (**10**)

To a solution of **9** (578 mg, 1.72 mmol) in DCM (5 mL), pyridine (410 μL, 5.1 mmol) and Tf_2_O (430 μL, 2.5 mmol) were added under an Ar atmosphere at −10 °C. The solution was stirred at −10 °C for 45 min, diluted with DCM, washed with cold aqueous saturated NaHCO_3_, dried (MgSO_4_), filtered, and concentrated. The residue was dissolved in DMF (4 mL), and NaNO_2_ (534 mg, 4.5 mmol, 4.5 equiv) was added under an Ar atmosphere at r.t. overnight. The solvent was removed under reduced pressure. The resulting residue was dissolved in CH_2_Cl_2_, washed with water, dried (MgSO_4_), filtered, concentrated, and purified by column chromatography (1:1 EtOAc–cyclohexane). Yield: 376 mg (65%, over two steps). *R*_f_ 0.35 (1:1 EtOAc–petroleum ether). [α]_D_ + 8.2 (*c* 1.0 in DCM). ^1^H NMR (300 MHz, CDCl_3_): δ 7.30–7.23 (m, 5H, CH_arom_), 5.79 (d, 1H, *J*_1,2_ = 5.9 Hz, H-1), 4.76 (d, 2H, ^2^*J*_H,H_ = 12.0 Hz, OCH_2_Ph), 4.63 (d, 1H, OCH_2_Ph), 4.38 (dd, 1H, *J*_6a,6b_ = 8.7 Hz, *J*_5,6a_ = 5.6 Hz, H-6a), 4.33 (t, 1H, *J*_5,6b_ = 8.9 Hz, H-6b), 4.18 (t, 1H, *J*_2,3_ = 6.4 Hz, H-2), 3.90 (m, 1H, H-5), 3.79 (bs, 1H, H-4), 3.41 (dd, 1H, *J*_3,4_ = 2.6 Hz, H-3), 2.38 (s, 1H, OH), 1.36 (s, 3H, CH_3_), 1.34 (s, 3H, CH_3_). ^13^C NMR (75.5 MHz, CDCl_3_): δ 156.9 (CO), 128.6–128.0 (C_arom_), 108.2 (CMe_2_), 80.4 (C-1), 78.7 (C-3), 73.5 (C-2), 71.8 (OCH_2_Ph), 66.3 (C-4), 63.4 (C-6), 52.3 (C-5), 27.9, 26.6 (CMe_2_). HRMS (ESI) calcd for [C_17_H_21_NO_6_Na]^+^, 358.1261; found, 358.1258.

#### (1*R*)-1,2,3,4-Tetra-*O*-acetyl-5*N*,6*O*-oxomethylidenegalactonojirimycin (**11**)

A solution of **10** (1.12 mmol) and 10% Pd/C (170 mg) in MeOH (9 mL) was hydrogenated under an atmospheric pressure of hydrogen. The mixture was stirred for 24 h, filtered through Celite, and concentrated. A solution of the crude (250 mg, 1.0 mmol) in 1:1 DCM–TFA (7.6 mL) was stirred at room temperature for 1 h. The solvent was eliminated under reduced pressure, co-evaporated several times with water, concentrated, and freeze-dried. Finally, further conventional acetylation afforded pure **11**. The spectroscopic and analytical data of compound **11** agree with those previously described.^69^

#### *N*α-Fluorenylmethyloxycarbonyl-*N*-dodecyl-l-serinamide (**14**)

To a solution of Fmoc–Ser–OH (3.2 g, 9.7 mmol) in THF (96 mL) were added HOBt (1.5 g, 11.1 mmol) and DCC (2 g, 11.1 mmol). The mixture was stirred until a DCU white precipitate appeared, and at that point, dodecylamine (1.8 g, 11.1 mmol) was added and stirred overnight. The crude mixture was filtered, concentrated, and purified by column chromatography using 1:1 EtOAc–cyclohexane as the eluent. Yield: 3.3 g (70%). *R*_f_ 0.50 (2:1 EtOAc–cyclohexane). [α]_D_ – 68.12 (*c* 1.0 in DCM). ^1^H NMR (300 MHz, CDCl_3_): δ 7.70–7.18 (m, 8H, CH_arom_), 6.47 (bt, 1H, *J*_NH,CH_ = 5.6 Hz, NH), 5.80 (d, 1H, *J*_NH,CH_ = 6.1 Hz, NH), 4.35 (d, 2H, *J*_H,H_ = 6.6 Hz, CH_2Fmoc_), 4.14 (t, 1H, *J*_H,H_ = 6.9 Hz, CH_Fmoc_), 4.06 (m, 2H, CH_2_O), 3.57 (m, 1H, CH), 3.15 (m, 2H, NHCH_2_), 1.85–0.80 (m, 23H, CH_2_, CH_2_CH_3_). ^13^C NMR (75.5 MHz, CDCl_3_): δ 170.8 (CO_amide_), 156.8 (CO_carbamate_), 143.6–120.0 (C_arom_), 67.3 (CH_2Fmoc_), 62.8 (CH), 55.0 (CH_2_O), 47.1 (CH_Fmoc_), 39.6 (NHCH_2_), 31.9–14.1 (CH_2_, CH_2_CH_3_). HRMS (ESI) calcd for [C_30_H_42_N_2_Na]^+^, 517.3037; found, 517.3029.

#### *N*α-Fluorenylmethyloxycarbonyl-*S*-trityl-*N*-dodecyl-l-cysteinamide (**15**)

To a solution of *N*-Fmoc–*S*-trityl-l-cysteine (3 g, 5.1 mmol) in THF (40 mL) were added HOBt (690 mg, 5.1 mmol) and DCC (1.1 g, 5.1 mmol). The mixture was stirred until a DCU white precipitate appeared, and at that point, dodecylamine (947 g, 5.1 mmol) was added and stirred overnight. The crude mixture was filtered, concentrated, and purified by column chromatography using 1:8 to 1:4 EtOAc–cyclohexane as the eluent. Yield: 3.8 g (97%). *R*_f_ 0.40 (1:4 EtOAc–cyclohexane). [α]_D_ + 4.68 (*c* 1.0 in DCM). ^1^H NMR (300 MHz, CDCl_3_): δ 7.69–7.10 (m, 23H, CH_arom_), 5.61 (t, 1H, *J*_NH,CH_ = 5.6 Hz, NH), 4.92 (d, 1H, *J*_NH,CH_ = 6.1 Hz, NH), 4.30 (d, 2H, *J*_H,H_ = 6.6 Hz, CH_2Fmoc_), 4.10 (t, 1H, *J*_H,H_ = 6.9 Hz, CH_Fmoc_), 3.69 (m, 1H, CH), 3.07 (m, 2H, NHCH_2_), 2.62 (dd, 1H, *J*_H,H_ = 15.0 Hz, *J*_H,H_ = 3.3 Hz, SCH_2_), 2.49 (dd, 1H, *J*_H,H_ = 15.0 Hz, *J*_H,H_ = 9.9 Hz, SCH_2_), 1.53–0.82 (m, 23H, CH_2_, CH_2_CH_3_). ^13^C NMR (75.5 MHz, CDCl_3_): δ 169.7 (CO_amide_), 155.9 (CO_carbamate_), 144.3–120.0 (C_arom_), 67.3 (CH_2Fmoc_), 66.9 (CH), 54.1 (CH_2_S), 47.1 (CH_Fmoc_), 39.6 (NHCH_2_), 31.9–14.1 (CH_2_, CH_2_CH_3_). HRMS (ESI) calcd for [C_49_H_56_N_2_O_3_SNa]^+^, 775.3904; found, 775.3895.

#### *N*α-Lauryl-*S*-trityl-*N*-dodecyl-l-cysteinamide (**16**)

Compound **15** (3.8 g, 5 mmol) was dissolved in THF (50 mL), and piperidine (10 mL) was added. The reaction was stirred for 15 min. Then, the solvent was removed under reduced pressure and co-evaporated with toluene (3 × 20 mL) to remove piperidine traces. The crude amine was dissolved in DCM (40 mL), and Et_3_N (850 μL, 5.9 mmol) and dodecyl chloride (1.3 mL, 5.9 mmol) were added. The mixture was then stirred overnight. After evaporation of the solvent, the crude product was purified by column chromatography using 1:4 EtOAc–cyclohexane as the eluent. Yield: 2 g (75%). [α]_D_ + 1.33 (*c* 1.0 in DCM). ^1^H NMR (300 MHz, CDCl_3_): δ 7.37–7.11 (m, 15H, CH_arom_), 5.92 (t, 1H, *J*_NH,CH_ = 5.6 Hz, NH), 5.69 (d, 1H, *J*_NH,CH_ = 6.1 Hz, NH), 4.01 (m, 1H, CH), 3.07 (m, 2H, NHCH_2_), 2.63 (dd, 1H, *J*_H,H_ = 15.0 Hz, *J*_H,H_ = 3.3 Hz, SCH_2_), 2.44 (dd, 1H, *J*_H,H_ = 15.0 Hz, *J*_H,H_ = 9.9 Hz, SCH_2_), 2.00 (m, 2H, CH_2_CO), 1.57–0.80 (m, 44H, CH_2_, CH_2_CH_3_). ^13^C NMR (75.5 MHz, CDCl_3_): δ 173.2, 169.9 (CO), 144.4–126.8 (C_arom_), 67.1 (CH), 52.0 (CH_2_S), 39.5 (NHCH_2_), 36.4 (CH_2_CO), 33.3–14.1 (CH_2_, CH_2_CH_3_). HRMS (ESI) calcd for [C_46_H_68_N_2_O_2_SNa]^+^, 735.4894; found, 735.4885.

#### *N*α-Lauryl-*N*-dodecyl-l-cysteinamide (**17**)

Compound **16** (600 mg, 0.83 mmol) was dissolved in 1:1 DCM–TFA (8 mL), and triisopropylsilane (510 μL, 2.4 mmol) was added. The reaction mixture was stirred for 2 h, and the solvent was evaporated under reduced pressure and co-evaporated with toluene (3 × 20 mL) to remove TFA traces. The resulting residue was purified by column chromatography (1:4 EtOAc–cyclohexane). Yield: 331 mg (85%). *R*_f_ 0.40 (1:2 EtOAc–cyclohexane). ^1^H NMR (300 MHz, CDCl_3_): δ 6.45 (m, 2H, NH), 4.50 (m, 1H, CH), 3.17 (m, 2H, CH_2_N), 2.94, 2.65 (m, 2H, SCH_2_), 2.16 (t, 2H, CH_2_CO), 1.61–0.81 (m, 44H, CH_2_, CH_2_CH_3_). ^13^C NMR (75.5 MHz, CDCl_3_): δ 173.4, 169.6 (CO), 54.1 (CH), 39.7 (CH_2_N), 36.5 (CH_2_CO), 31.9–14.0 (CH_2_, CH_2_CH_3_). HRMS (ESI) calcd for [C_27_H_54_O_2_N_2_SNa]^+^, 493.3798; found, 493.3793.

#### *N*α-Fluorenylmethyloxycarbonyl-*O*-(1*R*-2,3,4-tri-*O*-acetyl-5*N*,6*O*-oxomethylidene-galactonojirimycinyl)-*N*-dodecyl-l-serinamide (**18**)

To a solution of **11** (100 mg, 0.26 mmol) in dry DCM (5 mL), **14** (0.31 mmol) and BF_3_·OEt_2_ (130 μL, 1.05 mmol) were added, under an Ar atmosphere, at 0 °C. After 1 h, the mixture was diluted with DCM and washed with an aqueous 5% NaHCO_3_ solution. The resulting residue was purified by column chromatography (1:2 EtOAc–cyclohexane). Yield: 162 mg (65%). *R*_f_ 0.60 (2:1 EtOAc–cyclohexane). [α]_D_ + 33.7 (*c* 1.0 in DCM). ^1^H NMR (300 MHz, CDCl_3_): δ 7.95–7.28 (m, 12H, CH_arom_), 6.21 (bt, 1H, *J*_NH_,_CH2_ = 5.6 Hz, NHCH_2_), 5.65 (d, 1H, *J*_NH_,_CH_ = 7.8 Hz, NH_Fmoc_), 5.51 (d, 1H, *J*_1,2_ = 4.0 Hz, H-1), 5.42 (bt, 1H, *J*_3,4_ = 2.6 Hz, *J*_4,5_ = 2.2 Hz, H-4), 5.28 (dd, 1H, *J*_2,3_ = 10.9 Hz, H-3), 5.13 (dd, 1H, H-2), 4.07 (m, 4H, H-6a, OCH_2_CH, CH_2Fmoc_), 4.23 (m, 2H, H-5, CH_Fmoc_), 4.00 (td, 1H, *J*_5,6a_ = *J*_5,6b_ = 8.6 Hz, *J*_6a,6b_ = 5.9 Hz, H-6b), 3.79 (d, 2H, OCH_2_CH), 3.25 (m, 2H, NHCH_2_), 2.19, 2.10, 2.01 (3 s, 9H, CH_3_CO), 1.73–0.89 (m, 25 H, CH_2_, CH_2_CH_3_). ^13^C NMR (75.5 MHz, CDCl_3_): δ 170.2, 169.8 (CO ester), 155.9 (CO_carbamate_), 143.6–120.0 (C_arom_), 80.1 (C-1), 69.2 (OCH_2_CH), 67.8 (C-4, C-3), 67.2 (C-2, CH_2Fmoc_), 63.0 (C-6), 54.4 (OCH_2_CH), 50.6 (CH_Fmoc_), 47.0 (C-5), 39.8 (NHCH_2_), 31.9–14.1 (CH_3_CO, CH_2_, CH_2_CH_3_). ESIMS *m*/*z*: 830.38 [M + Na]^+^. HRMS (ESI) calcd for [C_43_H_57_O_12_N_3_Na]^+^, 830.3834; found, 830.3825.

#### *N*α-(9-Fluorenylmethoxycarbonyl)-*O*-(1*R*-2,3,4-tri-*O*-acetyl-5*N*,6*O*-oxomethylidene-nojirimycinyl)-*N*-dodecyl-l-serinamide (**19**)

To a solution of **13** (80 mg, 0.21 mmol) in DCM (5 mL), **14** (0.31 mmol) and BF_3_·OEt_2_ (130 μL, 1.05 mmol) were added, under an Ar atmosphere at 0 °C. After 1 h, the mixture was diluted with DCM (5 mL), washed with aqueous NaHCO_3_ (10 mL), dried with MgSO_4_, filtered, and concentrated. The resulting residue was purified by column chromatography (1:2 EtOAc–cyclohexane). Yield: 106 mg (63%). *R*_f_ 0.60 (2:1 EtOAc–cyclohexane). [α]_D_ + 37.5 (*c* 1.0 in DCM). ^1^H NMR (300 MHz, CDCl_3_): δ 7.70–7.20 (m, 12H, CH_arom_), 6.30 (t, 1H, *J*_NH_,_CH2_ = 5.6 Hz, NHCH_2_), 5.78 (d, 1H, *J*_NH_,_CH_ = 8.0 Hz, NH_Fmoc_), 5.48 (m, 2H, H-1, H-3), 4.94 (m, 1H, H-4), 4.92 (dd, 1H, *J*_2,3_ = 6.2 Hz, *J*_1,2_ = 4.6 Hz, H-2), 4.44 (m, 4H, H-6a, OCH_2_CH, CH_2Fmoc_), 4.25 (m, 2H, H-5, CH_Fmoc_), 3.98 (m, 1H, H-6b), 3.78 (m, 2H, OCH_2_CH), 3.27 (m, 2H, NHCH_2_), 2.09, 2.05, 2.04 (3 s, 9H, CH_3_CO), 1.55–0.89 (m, 25H, CH_2_, CH_2_CH_3_). ^13^C NMR (75.5 MHz, CDCl_3_): δ 169.9, 169.7, 169.0 (CO ester), 155.7 (CO_carbamate_), 143.6–120.0 (C_arom_),79.5 (C-1), 72.4 (OCH_2_CH), 70.2 (C-4), 69.1 (C-2), 68.9 (C-3), 67.3 (C-6), 67.0 (CH_2Fmoc_), 54.2 (OCH_2_CH), 51.7 (CH_Fmoc_), 47.0 (C-5), 39.9 (NHCH_2_), 31.9–14.1 (CH_3_CO, CH_2_, CH_2_CH_3_). ESIMS *m*/*z*: 830.38 [M + Na]^+^. HRMS (ESI) calcd for [C_43_H_57_O_12_N_3_Na]^+^, 830.3834; found, 830.3824.

#### *N*α-Lauryl-*O*-(1*R*-2,3,4-tri-*O*-acetyl-5*N*,6*O*-oxomethylidenegalactonojirimycinyl)-*N*-dodecyl-l-serinamide (**20**)

To a stirred solution of **18** (103 mg, 0.127 mmol) in DCM (1.5 mL), piperidine (127 μL) was added. After 1 h, the mixture was evaporated, and the crude product was used in the next step without further purification. To a solution of the crude product in DCM (1.7 mL), dodecyl chloride (27 μL, 0.127 mmol) and Et_3_N (72 μL, 0.508 mmol) were added, under an Ar atmosphere. The mixture was stirred for 16 h and concentrated. The resulting residue was purified by column chromatography (1:1 EtOAc–cyclohexane). Yield: 60 mg (70%, two steps). *R*_f_ 0.30 (1:1 EtOAc–cyclohexane). [α]_D_ + 40.8 (*c* 1.0 in DCM). ^1^H NMR (300 MHz, CDCl_3_): δ 6.39 (d, 1H, *J*_NH,CH_ = 8.2 Hz, NHCH), 6.34 (bt, 1H, *J*_NH,CH_ = 5.6 Hz, NHCH_2_), 5.51 (d, 1H, *J*_1_,_2_ = 4.0 Hz, H-1), 5.42 (bt, 1 H, *J*_3,4_ = 2.6 Hz, *J*_4,5_ = 2.2 Hz, H-4), 5.28 (dd, 1H, *J*_2,3_ = 10.7 Hz, H-3), 5.13 (dd, 1H, H-2), 4.61 (m, 1H, OCH_2_CH), 4.46 (t, 1H, *J*_5,6a_ = *J*_5,6b_ = 8.8 Hz, H-6a), 4.29 (m, 1H, H-5), 4.02 (dd, 1H, *J*_5,6b_ = 6.0 Hz, H-6b), 2.00 (d, 2H, ^2^*J*_H,H_ = 6.6 Hz, OCH_2_CH), 3.49 (m, 2H, COCH_2_), 3.25 (m, 2H, NHCH_2_), 2.19, 2.12, 2.01 (3 s, 9H, CH_3_CO), 1.64–0.89 (m, 48H, CH_2_, CH_2_CH_3_). ^13^C NMR (75.5 MHz, CDCl_3_): δ 173.4, 170.2, 169.8, 169.1 (CO_ester_), 156.1 (CO_carbamate_), 80.1 (C-1), 68.8 (C-4), 67.8 (C-3, OCH_2_CH), 67.2 (C-2), 63.1 (C-6), 52.3 (OCH_2_CH), 50.6 (C-5), 49.1 (COCH_2_), 39.8 (NHCH_2_), 36.5–14.1 (CH_3_CO, CH_2_, CH_2_CH_3_). ESIMS *m*/*z*: 790.48 [M + Na]^+^. HRMS (ESI) calcd for [C_40_H_69_O_11_N_3_Na]^+^, 790.4824; found, 790.4816.

#### *N*α-Lauryl-*O*-(1*R*-2,3,4-tri-*O*-acetyl-5*N*,6*O*-oxomethylidenenojirimycinyl)-*N*-dodecyl-l-serinamide (**21**)

To a stirred solution of **19** (106 mg, 0.131 mmol) in DCM (1.5 mL), piperidine (131 μL, 1 mL/mmol) was added. After 1 h, the mixture was evaporated, and the crude product was used in the next step without further purification. The crude product was dissolved in DMF (1.8 mL), and lauric acid (27 mg, 0.131 mmol), HBTU (99 mg, 0.262), and DIPEA (91 μL, 0.524 mmol) were added, under an Ar atmosphere. The mixture was stirred for 16 h and concentrated. The resulting residue was purified by column chromatography (1:1 EtOAc–cyclohexane).Yield: 65 mg (75%, two steps). *R*_f_ 0.40 (1:1 EtOAc–cyclohexane). [α]_D_ + 27.9 (*c* 1.0 in DCM). ^1^H NMR (300 MHz, CDCl_3_): δ 6.36 (d, 1 H, *J*_NH,CH_ = 7.8 Hz, NHCH), 6.29 (bt, 1H, *J*_NH,CH_ = 5.6 Hz, NHCH_2_), 5.46 (d, 1H, *J*_1_,_2_ = 4.7 Hz, H-1), 5.45 (m, 1H, H-3), 4.94 (t, 1H, *J*_4,5_ = 9.4 Hz, H-4), 4.91 (bdd, 1H, *J*_2,3_ = 9.9 Hz, H-2), 4.60 (m, 1H, OCH_2_CH), 4.49 (t, 1H, *J*_5,6a_ = *J*_5,6b_ = 8.5 Hz, H-6a), 4.27 (bdd, 1H, H-6b), 3.99 (m, 1H, H-5), 3.76 (m, 2H, OCH_2_CH), 3.28 (m, 2H, NHCH_2_), 2.24 (m, 2H, COCH_2_), 2.11, 2.06, 2.04 (3 s, 9H, CH_3_CO), 1.64–0.89 (m, 48H, CH_2_, CH_2_CH_3_). ^13^C NMR (75.5 MHz, CDCl_3_): δ 173.3, 169.9, 169.6, 169.2 (CO_ester_), 155.8 (CO_carbamate_), 79.6 (C-1), 72.5 (C-4), 70.2 (C-2), 68.9 (C-3), 68.8 (OCH_2_CH), 67.1 (C-6), 52.3 (OCH_2_CH), 51.8 (C-5), 39.8 (NHCH_2_), 36.5 (COCH_2_), 31.9–14.1 (CH_3_CO, CH_2_, CH_2_CH_3_). ESIMS *m*/*z*: 768.50 [M + H]^+^. HRMS (ESI) calcd for [C_40_H_69_O_11_N_3_Na]^+^, 790.4824; found, 790.4810.

#### *N*α-Lauryl-*O*-(1*R*-2,3,4-tri-*O*-acetyl-5*N*,6*O*-oxomethylidenegalactonojirimycinyl)-*N*-dodecyl-l-cysteinamide (**22**)

To a solution of **11** (20 mg, 0.05 mmol) and **17** (0.10 mmol), BF_3_·OEt_2_ (32 μL, 0.25 mmol) was added under an Ar atmosphere at 0 °C. After 1 h, the mixture was diluted with DCM (5 mL), washed with saturated NaHCO_3_ (3 × 10 mL), dried with MgSO_4_, filtered, and concentrated. The resulting residue was purified by column chromatography (1:1 EtOAc–cyclohexane). Yield: 27 mg (70%). *R*_f_ 0.40 (1:1 EtOAc–cyclohexane). [α]_D_ + 31.24 (*c* 1.0 in DCM). ^1^H NMR (500 MHz, CDCl_3_): δ 6.39 (m, 2H, NH), 5.86 (d, 1H, *J*_1_,_2_ = 3.9 Hz, H-1), 5.35 (bs, 1H, H-4), 5.08 (bd, 2H, H-2, H-3), 4.60 (dd, 1H, *J*_5,6a_ = *J*_5,6b_ = 8.6 Hz, H-6a), 4.57 (m, 1H, H-5), 4.42 (m, 1H, CH), 3.98 (dd, 1H, *J*_5,6b_ = 5.1 Hz, H-6b), 3.12 (m, 2H, NHCH_2_), 2.95 (dd, 1H, *J*_H,H_ = 15.0 Hz, *J*_H,H_ = 1.8 Hz, SCH_2_), 2.68 (dd, 1H, *J*_H,H_ = 15.0 Hz, *J*_H,H_ = 10.4 Hz, SCH_2_), 2.18 (m, 2H, CH_2_CO), 2.10, 2.02, 1.93 (3 s, 9H, CH_3_CO), 1.58–0.81 (m, 48H, CH_2_, CH_2_CH_3_). ^13^C NMR (125.7 MHz, CDCl_3_): δ 173.8, 170.1, 169.9, 169.7 (CO_ester_), 157.0 (CO_carbamate_), 68.5 (C-1), 67.4 (C-4), 67.2 (C-3), 63.5 (C-2), 60.6 (C-6), 52.9 (CH), 50.3 (C-5), 39.7 (NHCH_2_), 36.7–14.1 (CH_3_CO, CH_2_, CH_2_CH_3_). HRMS (ESI) calcd for [C_40_H_69_O_10_N_3_SNa]^+^, 806.4596; found, 806.4590.

#### *N*α-Lauryl-*O*-(1*R*-2,3,4-tri-*O*-acetyl-5*N*,6*O*-oxomethylidenenojirimycinyl)-*N*-dodecyl-l-cysteinamide (**23**)

To a solution of **13** (20 mg, 0.05 mmol) and **17** (0.10 mmol), BF_3_·OEt_2_ (32 μL, 0.25 mmol) was added under an Ar atmosphere at 0 °C. After 1 h, the mixture was diluted with DCM (5 mL), washed with saturated NaHCO_3_ (3 × 10 mL), dried with MgSO_4_, filtered, and concentrated. The resulting residue was purified by column chromatography (1:1 EtOAc–cyclohexane). Yield: 27 mg (70%). *R*_f_ 0.50 (1:1 EtOAc–cyclohexane). [α]_D_ + 16.24 (*c* 1.0 in DCM). ^1^H NMR (500 MHz, CDCl_3_): δ 6.39 (d, 1H, *J*_NH,CH_ = 9.0 Hz, NHCH), 6.38 (bt, 1H, *J*_NH,CH_ = 5.6 Hz, NHCH_2_), 5.73 (d, 1H, *J*_1,2_ = 5.9 Hz, H-1), 5.28 (t, 1H, *J*_2,3_ = *J*_3,4_ = 10.4 Hz, H-3), 4.86 (t, 1H, *J*_4,5_ = 9.4 Hz, H-4), 4.85 (dd, 1H, H-2), 4.61 (t, 1H, *J*_5,6a_ = *J*_5,6b_ = 8.6 Hz, H-6a), 4.42 (m, 1H, CH), 4.30 (bdd, 1H, H-5), 4.24 (m, 1H, H-6b), 3.11 (m, 2H, NHCH_2_), 2.93 (dd, 1H, *J*_H,H_ = 15.0 Hz, *J*_H,H_ = 3.3 Hz, SCH_2_), 2.72 (dd, 1H, *J*_H,H_ = 15.0 Hz, *J*_H,H_ = 9.9 Hz, SCH_2_), 2.18 (m, 2H, COCH_2_), 2.01, 1.99, 1.96 (3 s, 9H, CH_3_CO), 1.57–0.81 (m, 48H, CH_2_, CH_2_CH_3_). ^13^C NMR (125.7 MHz, CDCl_3_): δ 173.8, 170.0, 169.7, 169.4 (CO_ester_), 156.8 (CO_carbamate_), 72.6 (C-4), 70.3 (C-2), 69.5 (C-3), 67.3 (C-6), 60.2 (C-1), 53.1 (CH), 51.2 (C-5), 39.7 (NHCH_2_), 36.7 (COCH_2_), 31.9–14.1 (CH_3_CO, CH_2_, CH_2_CH_3_). HRMS (ESI) calcd for [C_40_H_69_N_3_O_10_SNa]^+^, 806.4596; found, 806.4588.

### Biology

α-GalCer (αGC, KRN7000) was purchased from Funakoshi (Tokyo, Japan). RPMI 1640, DMEM, fetal bovine serum (FBS), newborn calf serum (NBCS), and red blood cell (RBC) lysis buffer were purchased from Gibco, Thermo Fisher Scientific. Ovalbumin (OVA) was purchased from Worthington Biochemicals (LS003049) (Freehold, NJ, USA). OVA_257-264_ peptide was purchased from InvivoGen (San Diego, CA, USA).

#### In Vitro Culture of Human PBMCs

Human PBMCs from normal volunteers were collected from whole blood and isolated by separation over a histopaque-1077 (Sigma) density gradient. All donors provided informed consent, and the respective institutional review boards approved all experimental protocols. Supernatants were collected for ELISA analysis.

#### In Vitro Stimulation of Splenocytes, 293 Cells, and Mouse iNKT Hybridoma DN3A4–1.2 Cells

Stock solutions of the compounds were prepared in DMSO at a 20 mM concentration, and aliquots of these solutions were added to PBS to reach the desired target concentrations. The final proportion of DMSO was <5% in all cases. Mouse splenocytes (1 × 10^6^ cells/well) were isolated and stimulated with the glycolipids in 96-well plates for 48 h. HEK-293 (293), 293/hTLR2, 293/hMD2–CD14, and 293/hTLR4–MD2–CD14 cells were from InvivoGen (San Diego, CA, USA). The cells (2 × 10^5^ cells/well) were stimulated in the presence of the indicated glycolipid mimetic in 96-well plates for 24 h. DN3A4–1.2, A20, and A20–CD1d cells were kindly provided by Dr. Mitchell Kronenberg (La Jolla Institute, CA) and were maintained in RPMI 1640 medium. DN3A4–1.2 cells (1 × 10^5^ cells/well) were stimulated by co-culturing with A20 or A20–CD1d cells (5 × 10^4^ cells/well) in the presence of the indicated glycolipids in 96-well plates for 24 h.

#### Mice

Eight- to ten-week-old C57BL/6 and BALB/c female mice were purchased from the National Laboratory Animal Center (Taipei, Taiwan). OT-1 mice were kindly provided by Dr. Liao Nan-Shih (Academia Sinica, Taiwan). All animals were housed under specific pathogen-free conditions. All protocols were approved by the Academia Sinica Institutional Animal Care and Use Committee (IACUC), and all experiments were performed according to the guidelines of the IACUC and adhered to the guidelines on animal studies outlined in the ACS Ethical Guidelines.

### OT-1 Adjuvancy Assay

Carboxyfluorescein succinimidyl ester (CFSE)-labeled splenocytes from OT-1 mice were intravenously (2 × 10^7^/mice) transferred to B6 mice. 2 h later, the mice were immunized by i.p. injection of OVA_257–264_ peptide (0.1 μg/mice) with indicated compounds (20 μg/g mouse weight). Two days after the immunization, the proliferation of OT-1 CD8 T cells was analyzed by flow cytometry.

#### Flow Cytometry

Single-cell suspensions were stained with the fixable viability dye eFluor 780 (eBioscience) for 30 min at 4 °C, and Fc receptors were blocked with an anti-CD16/32 (BioLegend) blocking antibody prior to surface staining with monoclonal antibodies. Antibodies used for surface staining are as listed: mouse: CD45 (30-F11, BioLegend), lineage (Lin) markers [CD3 (145-2C11, BioLegend), CD19 (6D5, BioLegend), CD11b (M1/70, BioLegend), CD11c (N418, BioLegend)], and CD86 (GL-1, BioLegend). For CFSE staining, cells were stained with 5 μM CFSE according to the manufacturer’s protocol. Data were acquired on LSR II (BD Biosciences) and analyzed with FlowJo v. 10.1 software (TreeStar).

### OVA Challenge Model

For the OVA challenge, mice were sensitized intraperitoneally with 10 μg of OVA and 2% alhydrogel adjuvant (InvivoGen). Mice were challenged three times with 10 μg of OVA on day 14 post-sensitization, either daily or every other day. Mice were sacrificed the day after the last OVA challenge.

#### Measurement of AHR

Mice were anesthetized with 100 mg per kg body weight pentobarbital (Sigma-Aldrich), tracheotomized, and mechanically ventilated via the FinePointe RC system (Buxco Research Systems, Wilmington, NC). Lung function was assessed by measuring lung resistance and dynamic compliance in response to increasing doses of aerosolized methacholine (1.25–40 mg/mL, Sigma-Aldrich).

#### Collection and Analysis of BALF

Upon exposure of the trachea, the lungs were lavaged twice with 1 mL of PBS supplemented with 2% fetal calf serum (FCS). BALF was pooled, and BAL cells were pelleted by centrifugation and fixed onto cytospin slides. The slides were stained with Diff-Quik solution (Polysciences Inc), and a BAL differential cell count was performed.

#### Lung Cell Isolation

Whole lungs were flushed by PBS (supplemented with 2% FCS) through the right ventricle and minced prior to incubation in 3 mL of the DMEM medium with 0.1% (vol/vol) DNase I (Worthington Biochemicals) and 1.6 mg/mL collagenase IV (Worthington Biochemicals) for 40 min at 37 °C. Tissues were filtered through a 100 μm mesh to obtain single-cell suspensions. Red blood cells were removed by incubation in ACK lysing buffer (GIBCO) for 5 min at 25 °C. Single-cell suspensions were resuspended in the appropriate buffer for further processing.

#### ELISA

Cytokines (mouse IL-2, IL-4, IL-12p40, IL-13, and IFN-γ; human IL-6, IL-8, and IFN-γ) in the supernatants of cell cultures, as well as in the lungs, serum, and BAL fluid of mice were analyzed with ELISA kits from Biolegend, with the exception of mouse IL-13 (eBioscience). For determination of cytokine concentrations in vivo, the lungs were flushed and minced thoroughly prior to sonication with the Bioruptor Plus sonicator (Diagenode) in RIPA buffer. Lung protein lysates were obtained by centrifugation. For determination of cytokine concentrations in vitro, supernatants from treated cells were collected after centrifugation and analyzed by capture ELISA according to the manufacturer’s protocol.

#### Real-Time PCR

Total RNA from cultured cells and tissues was extracted using the Direct-zol MiniPrep (Zymo Research, Irvine, CA), and 2 μg of cDNA was synthesized using the high-capacity cDNA reverse transcription kit (Applied Biosystems, Foster City, CA). The expression levels of IL-4 and CD86 were measured by real-time qPCR using SYBR green on a TOptical 96 real-time PCR thermal cycler (Biometra, Analytik Jena, Germany).

### Statistics

Data were analyzed with GraphPad Prism 6 software (GraphPad Prism software, San Diego, CA). Statistical analysis was determined using the two-way analysis of variance (ANOVA) or the Student’s two-tailed *t*-test. All data were expressed as mean ± SEM, and *p* values of <0.05 were considered statistically significant.

## Supporting Information Available

B6 mouse splenocytes and HEK-293 cell viability data (MTT assay) upon exposure to compounds **1–4**; flow cytometry analysis of adjuvant (compounds **1** or **2**)-promoted OT-1-derived CD8^+^ T cell proliferation; ^1^H and ^13^C NMR spectra; and HPLC purity (PDF)
Molecular formula strings (CSV)

## Abbreviations

Ac_2_O: acetic anhydride
AHR: airway hyperreactivity
BALF: bronchoalveolar fluid
BMDCs: bone marrow-derived dendritic cells
DAMPS: danger-associated molecular patterns
DCs: dendritic cells
CD: cluster of differentiation
DIPEA: *N*,*N*-diisopropylethylamine
αGarlCer: α-galactosylceramide
HBC: hepatitis B virus
HEK: human embryonic kidney cells
HPCs: hematopoietic cells
HPV: human papillomavirus
IFN: interferon
sp^2^-IGL: sp^2^-iminoglycolipid
IL: interleukin
LPS: lipopolysaccharide
MAPK: mitogen-activated protein kinase
MD-2: myeloid differentiation factor 2
MS: molecular sieves
MyD88: myeloid differentiation primary response 88 protein
MPL: monophosphoryl lipid A
iNKT: invariant natural killer cells
OGJ: 5*N*,6*O*-oxomethylidene-galactonojirimycin
ONJ: 5*N*,6*O*-oxomethylidene-nojirimycin
PAMPs: pathogen associated molecular patterns
PTSA: *p*-toluenesulfonic acid
quant: quantitative
OVA: ovalbumin
SACs: structural airway cells
TCR: T-cell receptor
Th: T helper
TRIF: TIR-domain-containing adapter-inducing interferon-β
TLR: toll-like receptor
