# Supplementary material for: Serine-/Cysteine-Based sp2-Iminoglycolipids as Novel TLR4 Agonists: Evaluation of Their Adjuvancy and Immunotherapeutic Properties in a Murine Model of Asthma
Source: J Med Chem. 2023 Mar 23;66(7):4768–83. doi: 10.1021/acs.jmedchem.2c01948 (PMC10108363; doi:10.1021/acs.jmedchem.2c01948)
Supplement: Supplementary file 1 — jm2c01948_si_001.pdf [file jm2c01948_si_001.pdf]

Supporting Information

Serine-/Cysteine-Based sp^2-Iminoglycolipids as Novel TLR4 Agonists: Evaluation of Their Adjuvancy and Immunotherapeutic Properties in a Murine Model of Asthma

Manuel González-Cuesta,[a†] Alan Chuan-Ying Lai,[b†] Po-Yu Chi,[b] I-Ling Hsu,[b] Nien-Tzu Liu,[b] Ko-Chien Wu,[b] José M. García Fernández,[c] Ya-Jen Chang,[b,d] Carmen Ortiz Mellet[a]**

[a] Department of Organic Chemistry, Faculty of Chemistry, University of Seville, E-41012 Seville, Spain.

[b] Institute of Biomedical Sciences, Academia Sinica, Nankang, Taipei 115, Taiwan.

[c] Instituto de Investigaciones Químicas (IIQ), CSIC – Universidad de Sevilla, Américo Vespucio 49, E-41092 Sevilla, Spain.

[d] Institute of Translational Medicine and New Drug Development, China Medical University, Taichung 404, Taiwan.

[†]These authors contributed equally to this work.

* Corresponding authors: E-mail: mellet@us.es, yajchang@ibms.sinica.edu.tw

Contents **Page(s)**

B6 Mouse splenocytes and HEK-293 cell viability data (MTT assay) upon exposure to compounds **1-4**. S2

Flow cytometry analysis of $CD8^+$ T cells proliferation S3

^1H and ^{13}C NMR spectra of new compounds S4-S22

HPLC traces of final compounds S23-S24

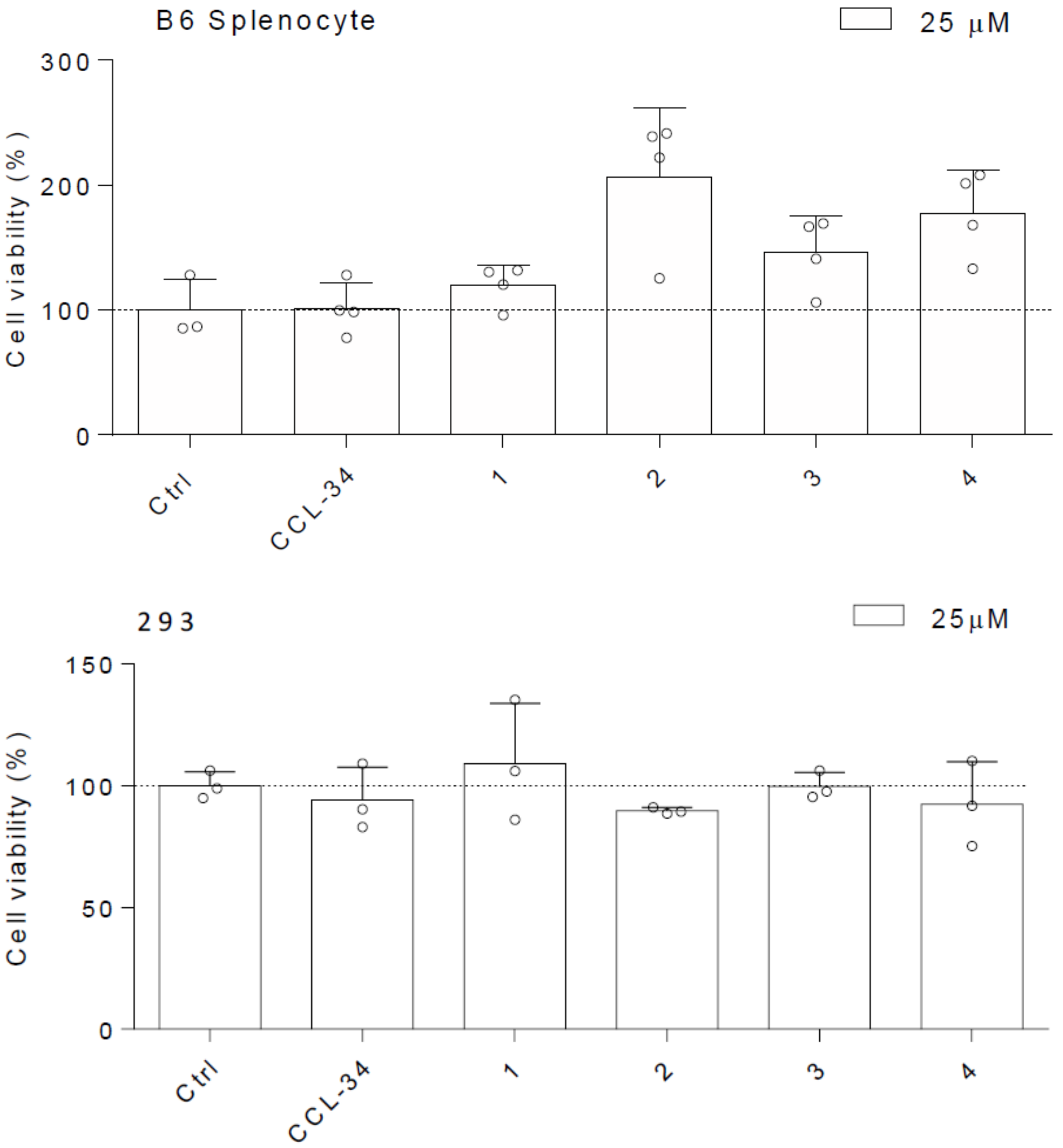

Figure S1. Treatments with compounds **1-4** do not cause reduced viability in B6 mouse splenocytes and HEK-293 cells. MTT assay was performed on B6 mouse splenocytes (upper panel) and HEK-293 cells (lower panel) post treatments with compounds **1-4** to determine the viability of cells after prolonged exposure to test compounds.

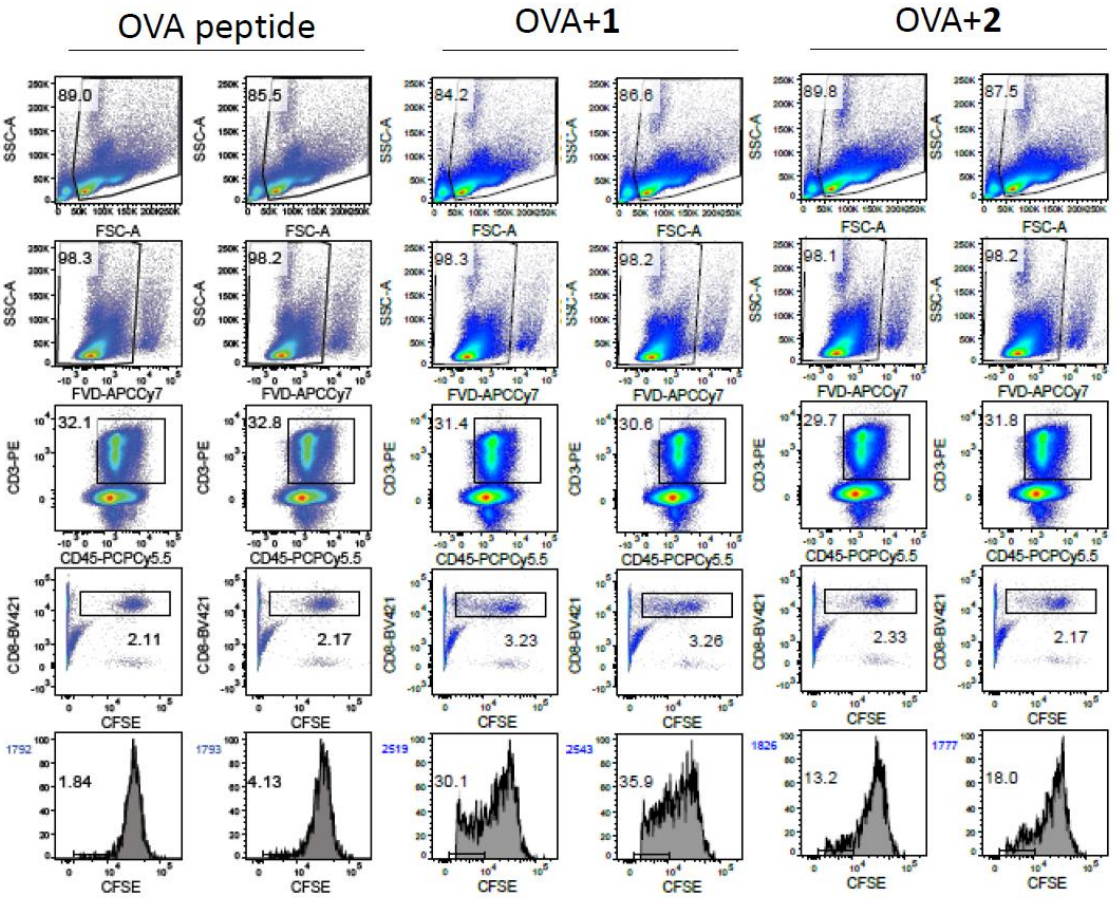

Figure S2. Flow cytometry analysis of adjuvant (compound 1 or 2)-promoted OT-1-derived CD8+ T cells proliferation. Representative data of flow cytometry analysis of experiment described in the main text, **Figure 6E, 6F**. CFSE positive $CD45^{+}CD3^{+}CD8^{+}$ T cell populations were gated and quantified from mice administered with different compounds in combination with $OVA_{257\text{-}264}$.

^{1}H and [13]C Spectra of new compounds

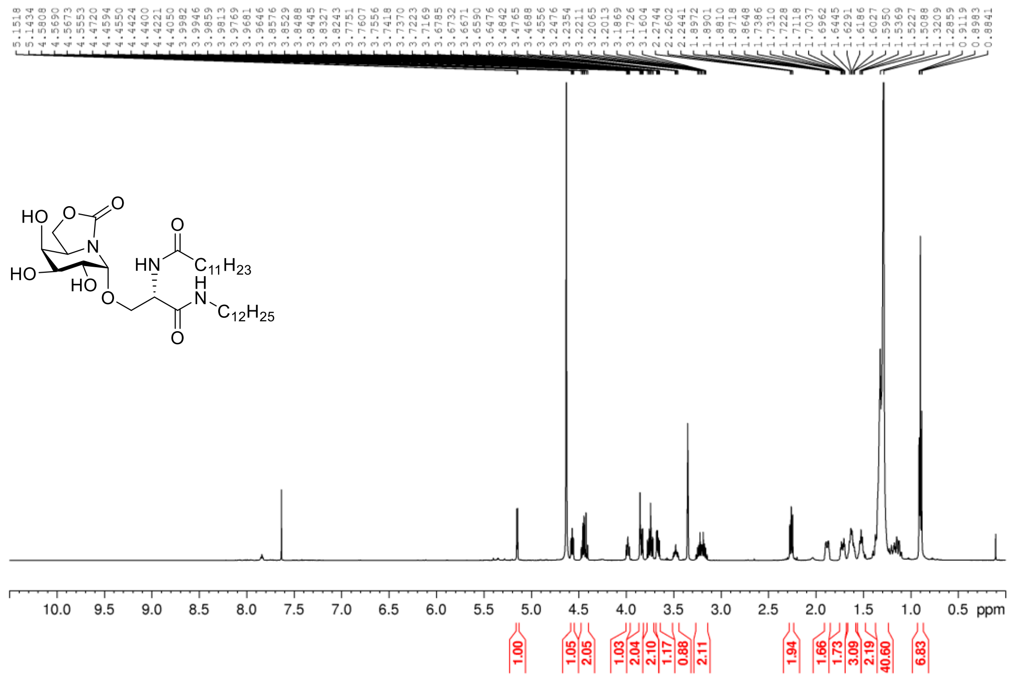

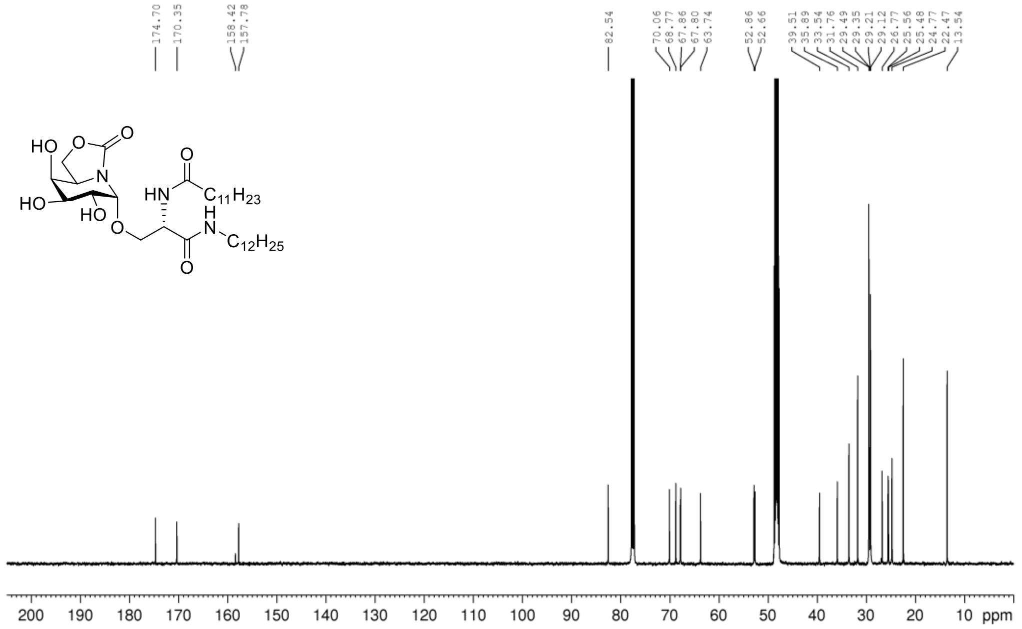

Figure S3. ^{1}H and ^{13}C NMR spectra (500 MHz, 125.5 MHz, 1:1 CD_3OD-$CDCl_3$) of **1**.

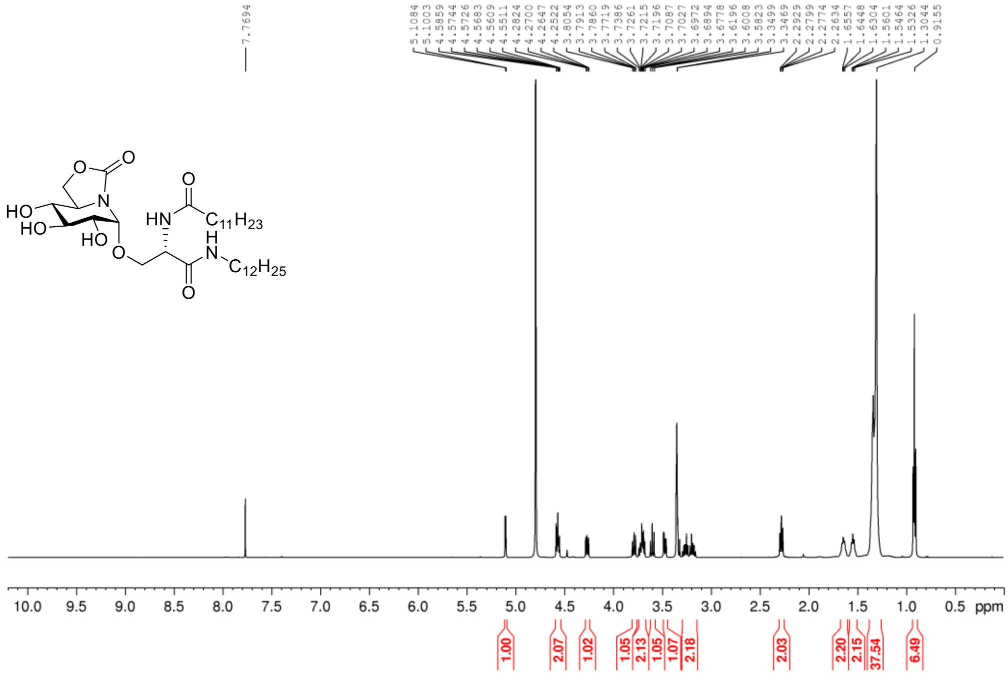

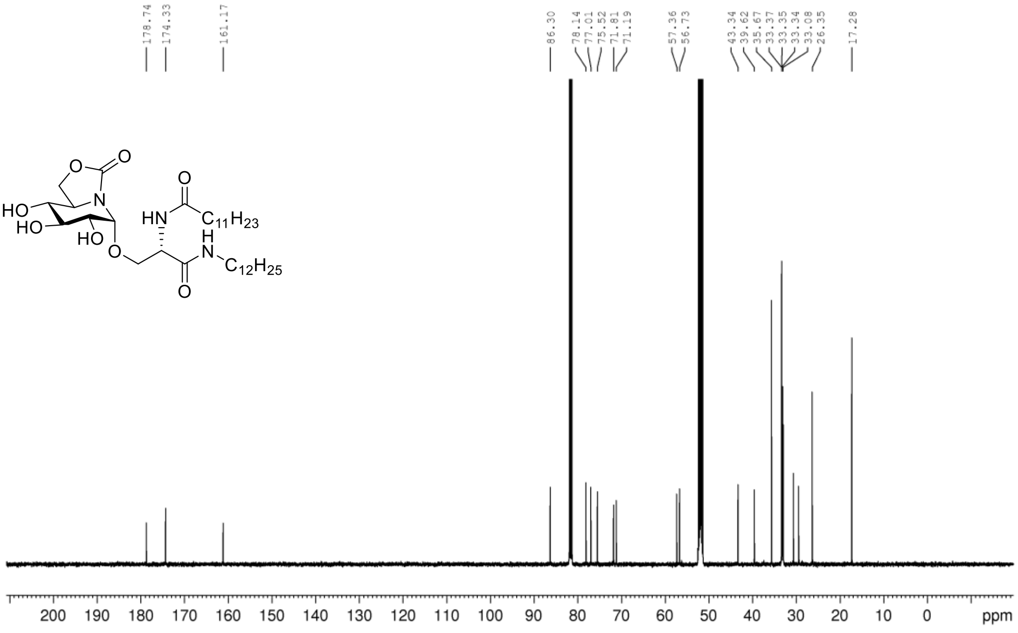

Figure S4. ^{1}H and ^{13}C NMR spectra (500 MHz, 125.5 MHz, 1:1 CD_3OD-$CDCl_3$) of **2**.

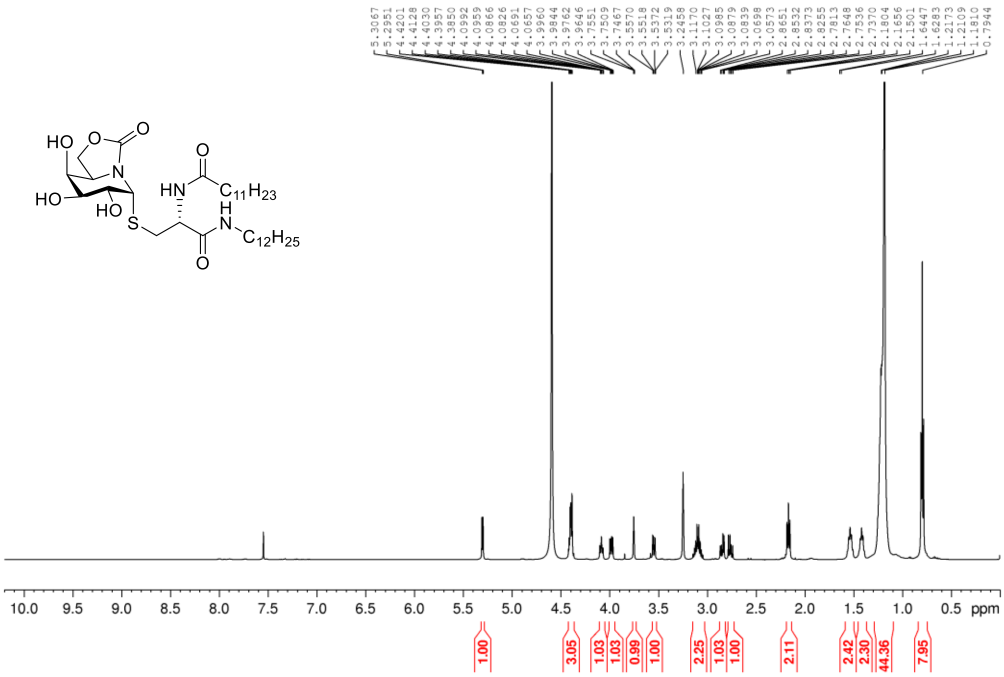

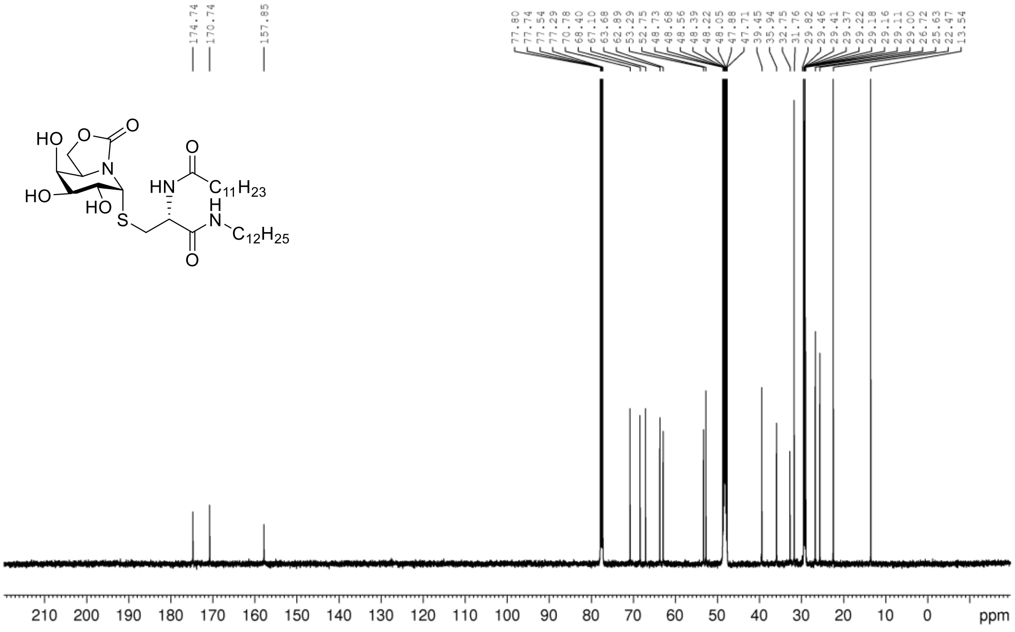

Figure S5. ^{1}H and ^{13}C NMR spectra (500 MHz, 125.7 MHz, 1:1 CD_3OD-$CDCl_3$) of **3**.

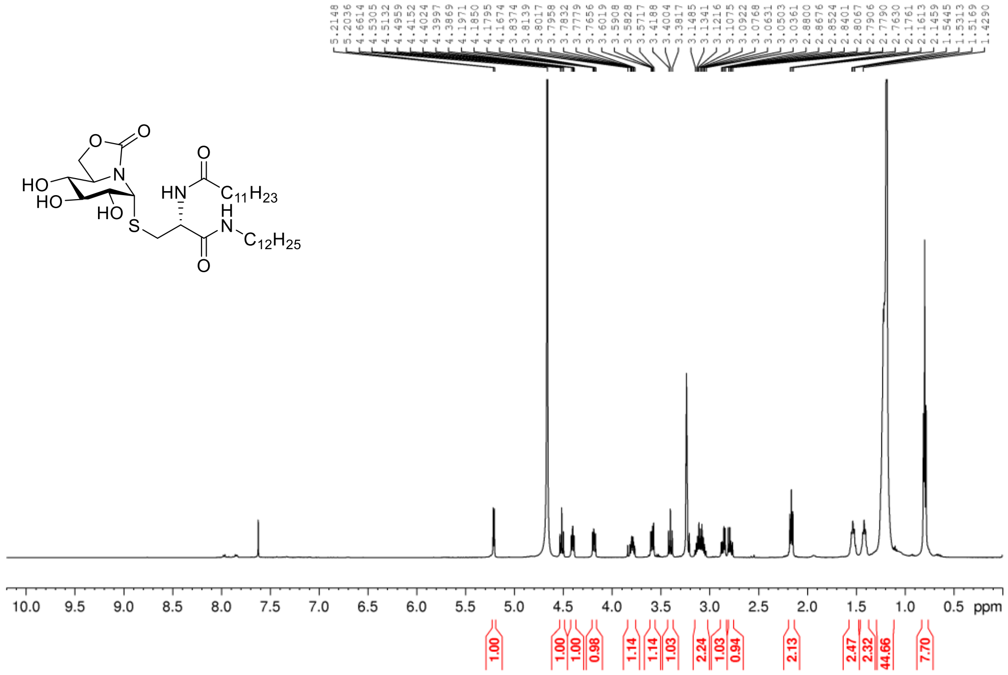

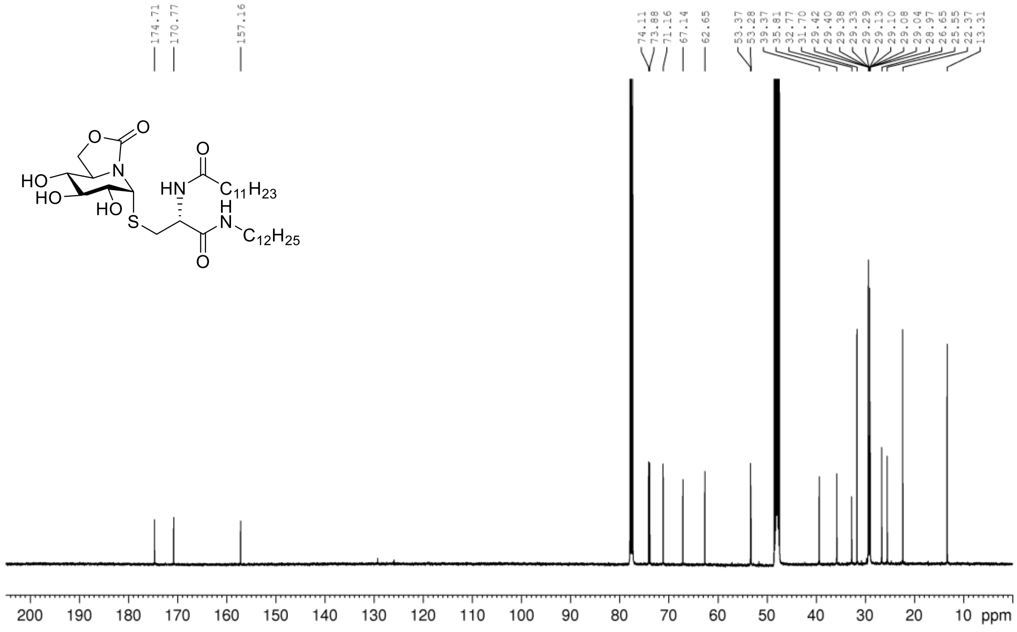

Figure S6. ^{1}H and ^{13}C NMR spectra (500 MHz, 125.7 MHz, 5:1 CD_3OD-$CDCl_3$) of **4**.

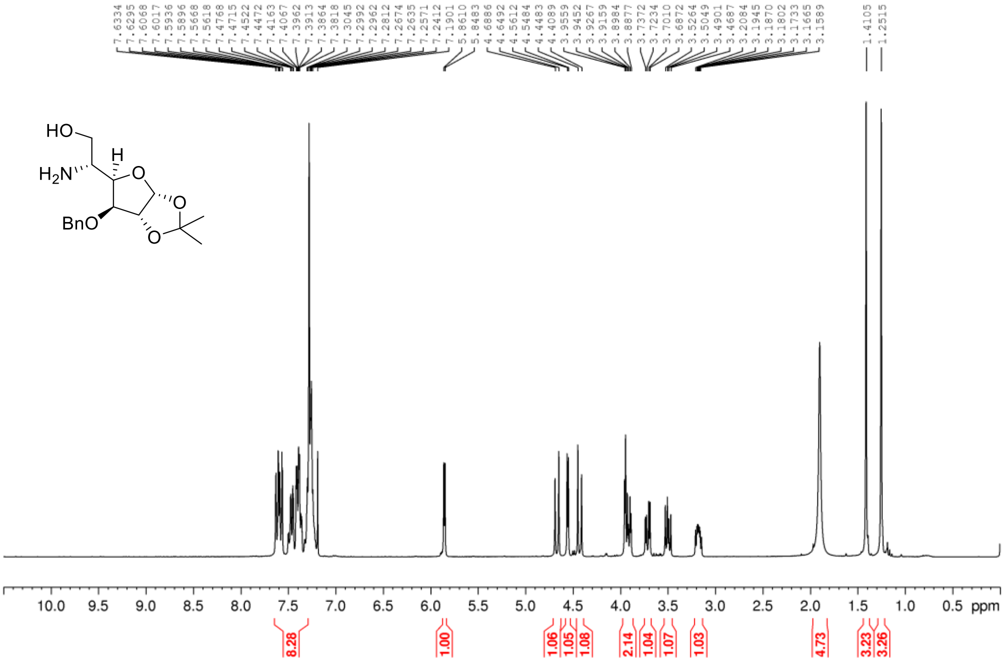

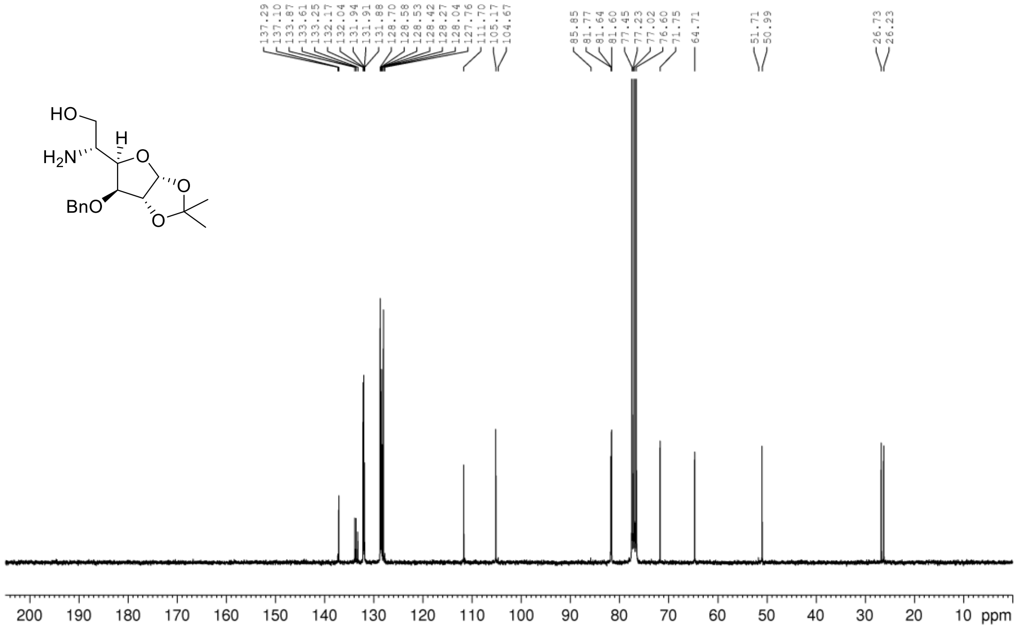

Figure S7. ^{1}H and ^{13}C NMR spectra (300 MHz, 75.5 MHz, $CDCl_3$) of **6**.

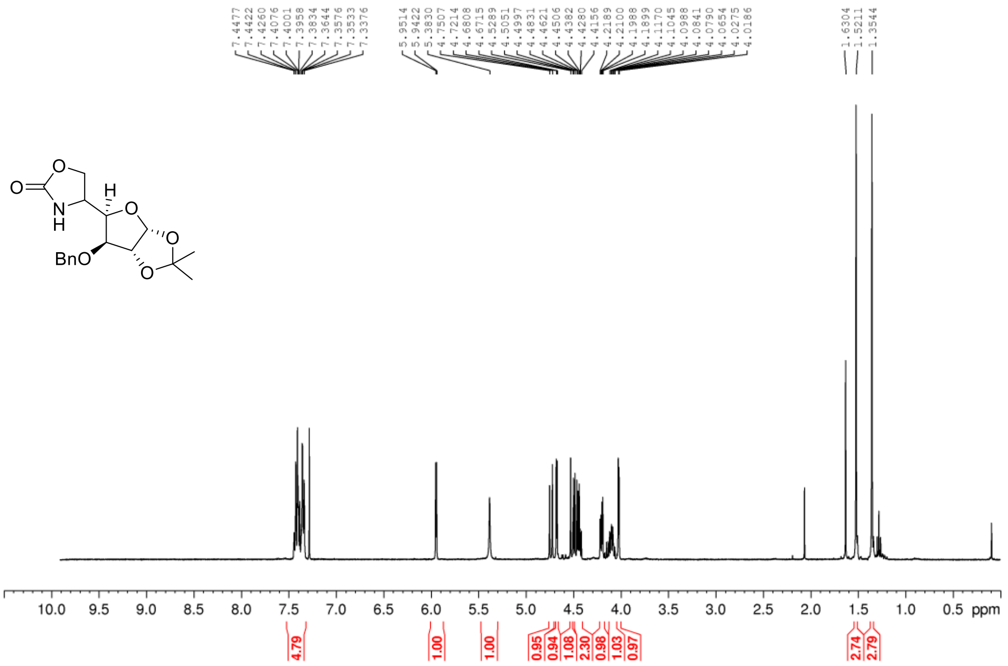

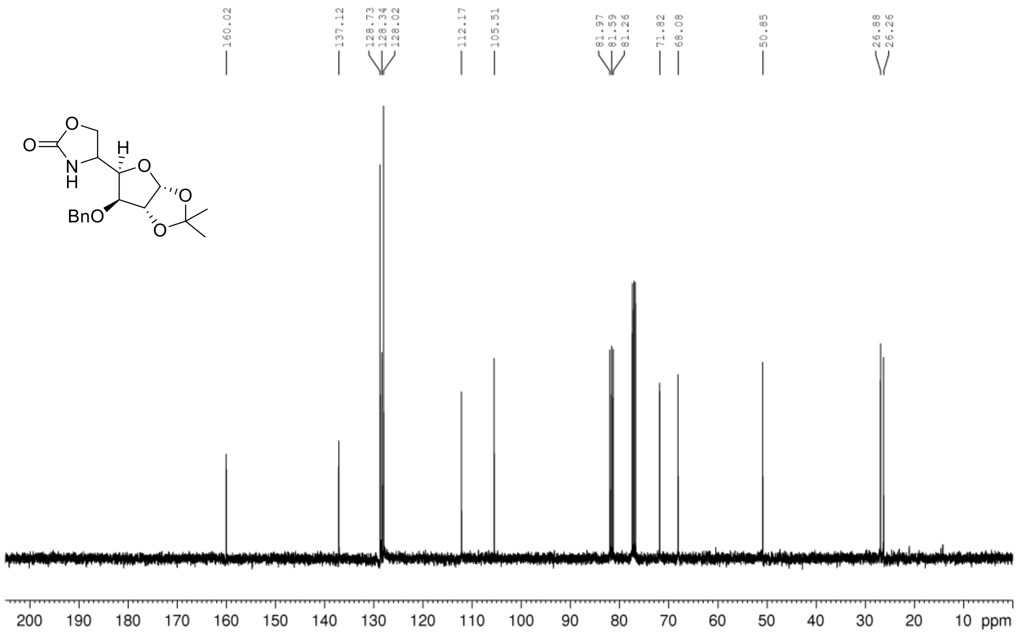

Figure S8. ^{1}H and ^{13}C NMR spectra (300 MHz, 75.5 MHz, $CDCl_3$) of **7**.

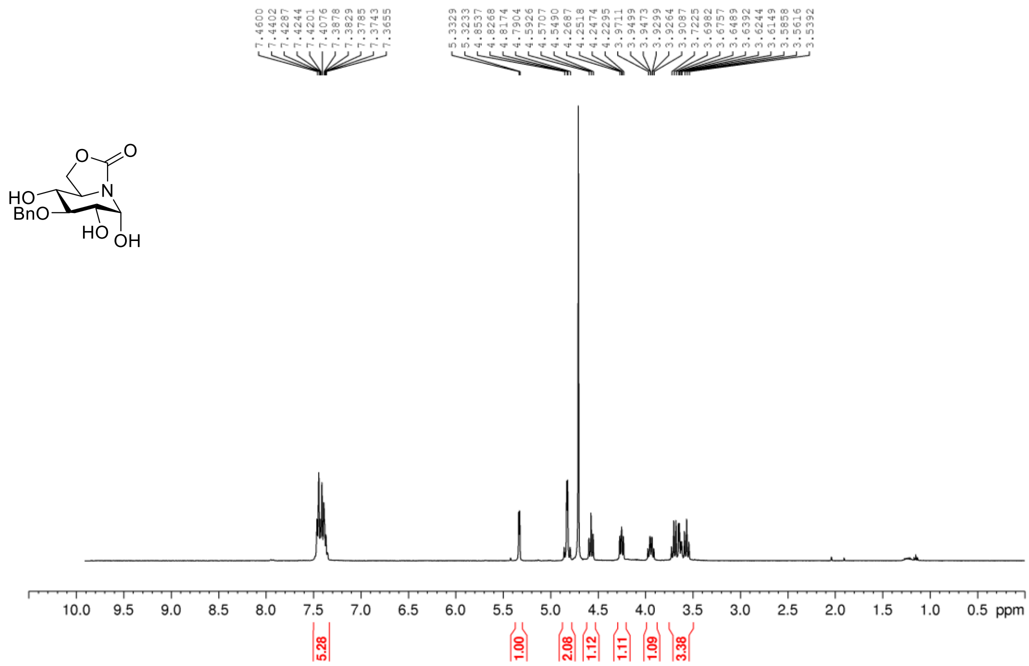

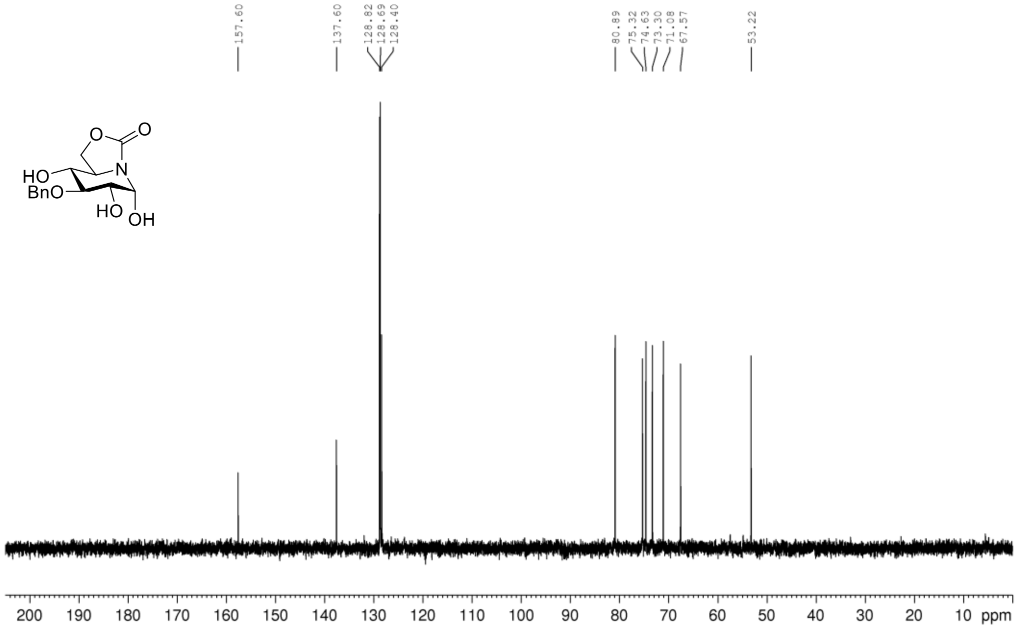

Figure S9. ^{1}H and ^{13}C NMR spectra (400 MHz, 100 MHz, $CDCl_3$) of **8**.

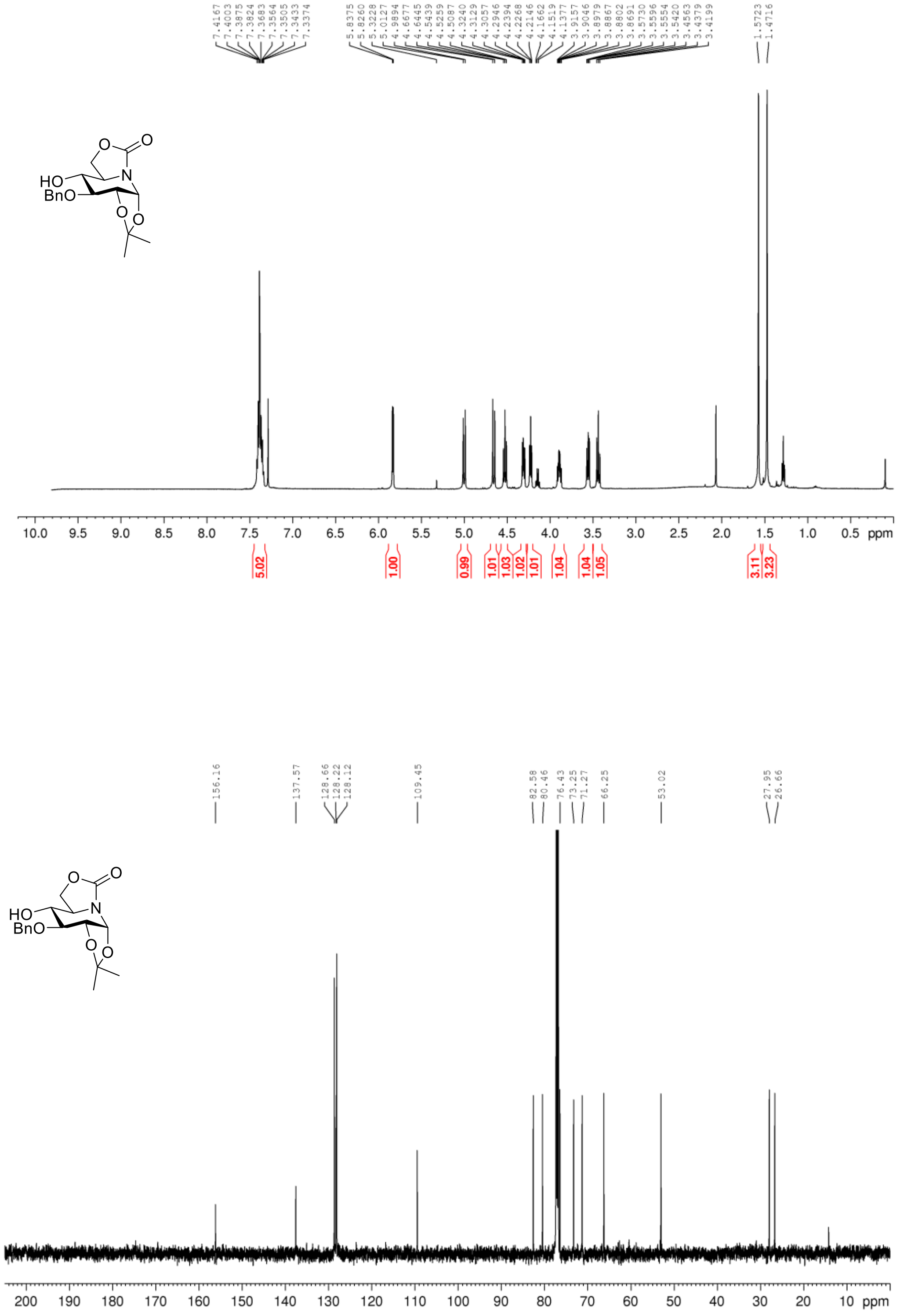

Figure S10. ^{1}H and ^{13}C NMR spectra (300 MHz, 75.5 MHz, $CDCl_3$) of **9**.

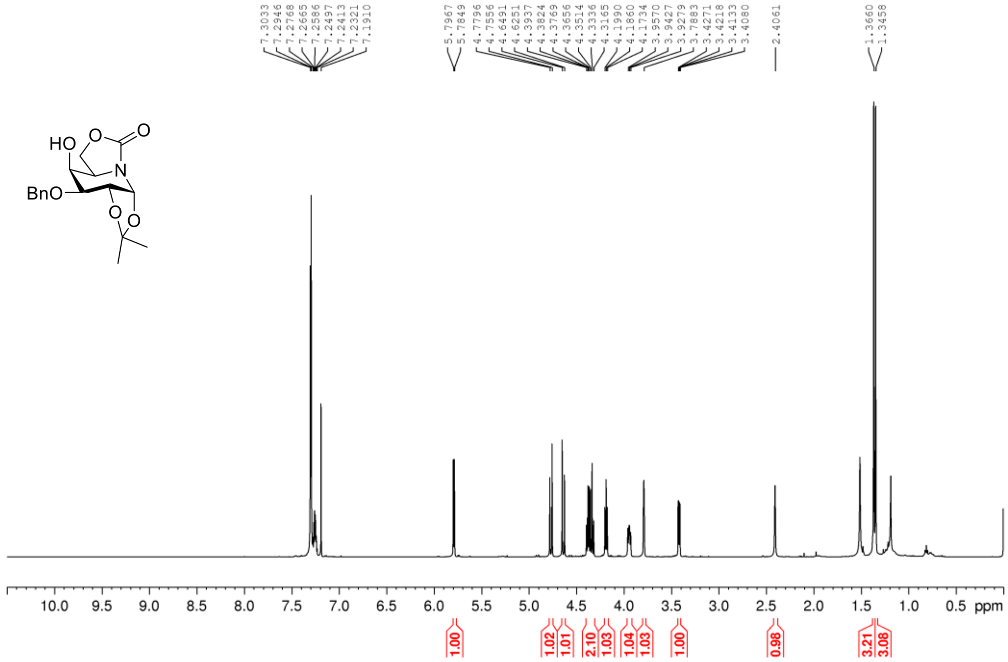

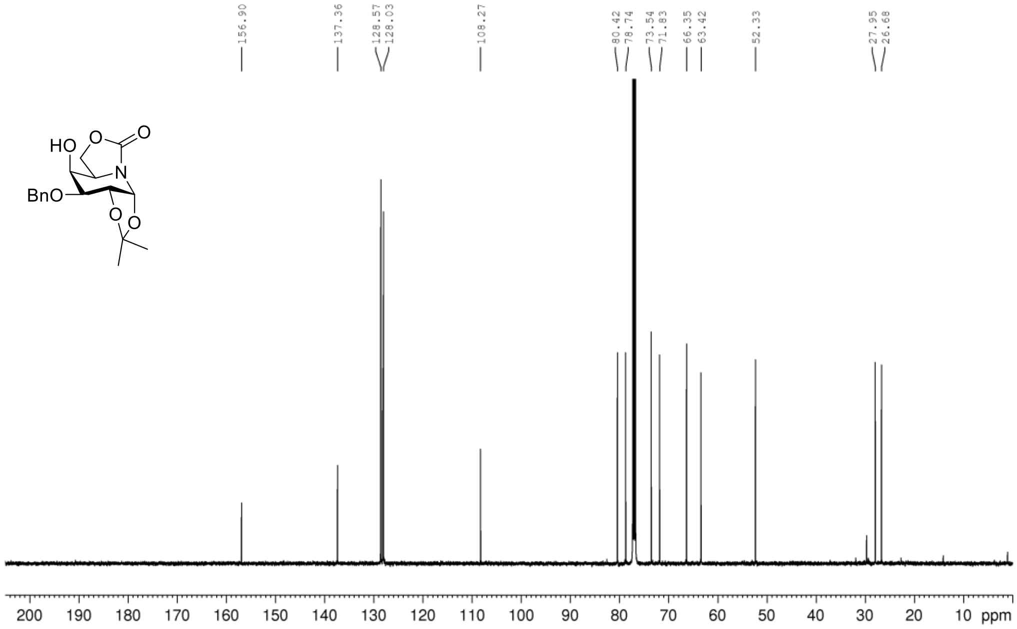

Figure S11. ^{1}H and ^{13}C NMR spectra (300 MHz, 75.5 MHz, $CDCl_3$) of **10**.

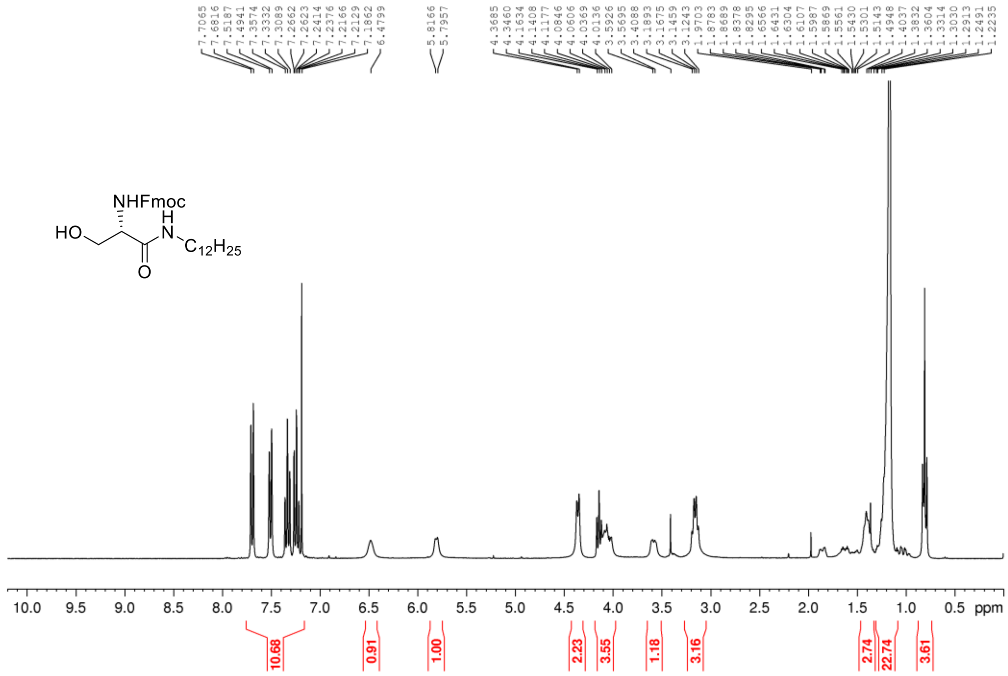

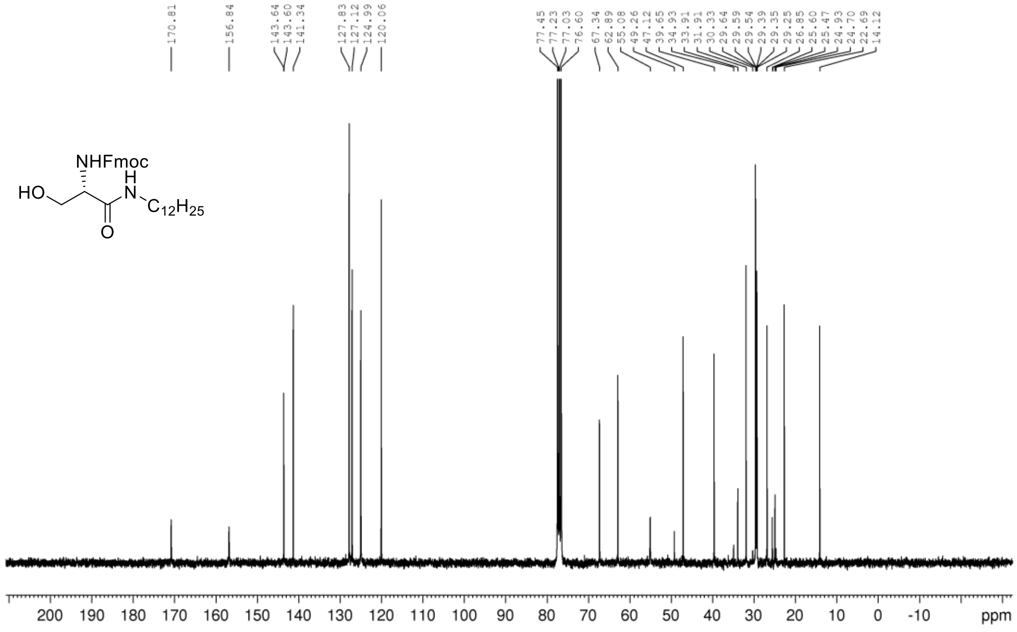

Figure S12. ^{1}H and ^{13}C NMR spectra (300 MHz, 75.5 MHz, $CDCl_3$) of **14**.

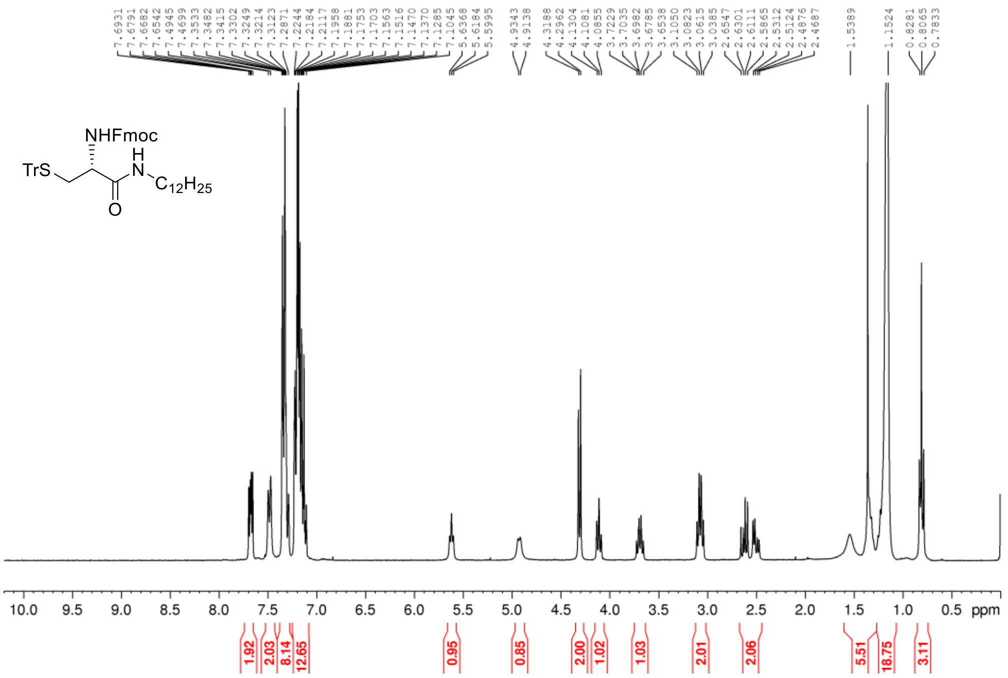

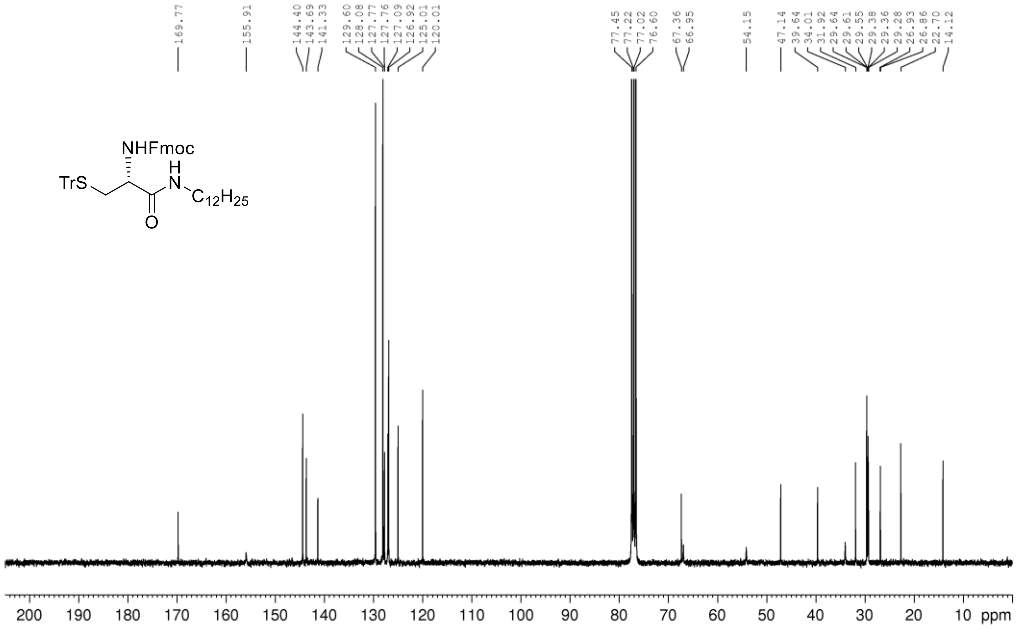

Figure S13. ^{1}H and ^{13}C NMR spectra (300 MHz, 75.5 MHz, $CDCl_3$) of **15**.

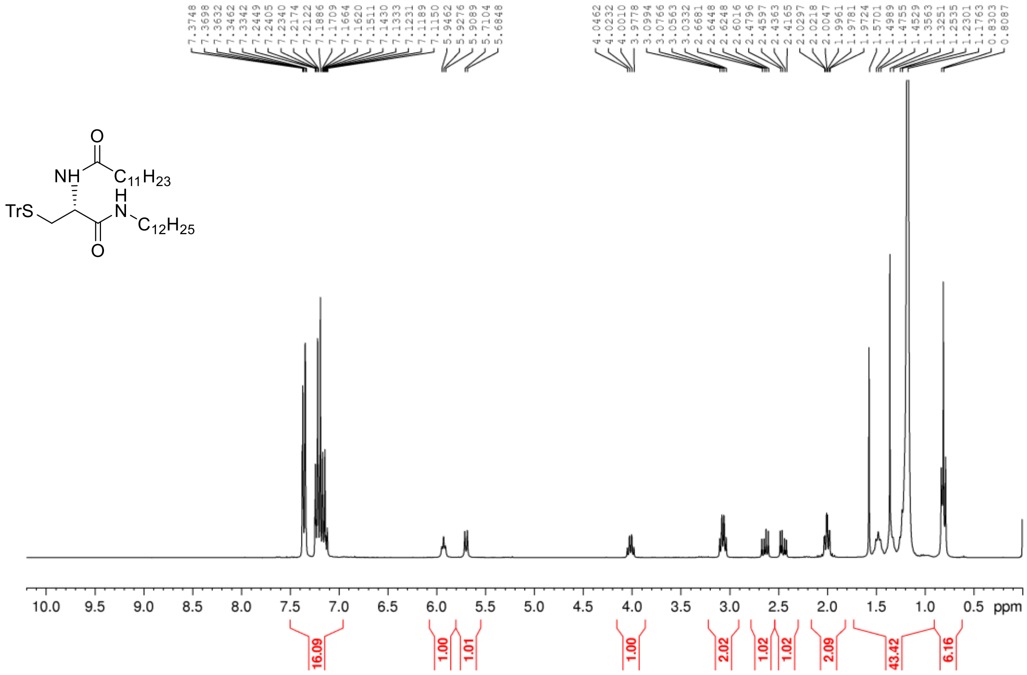

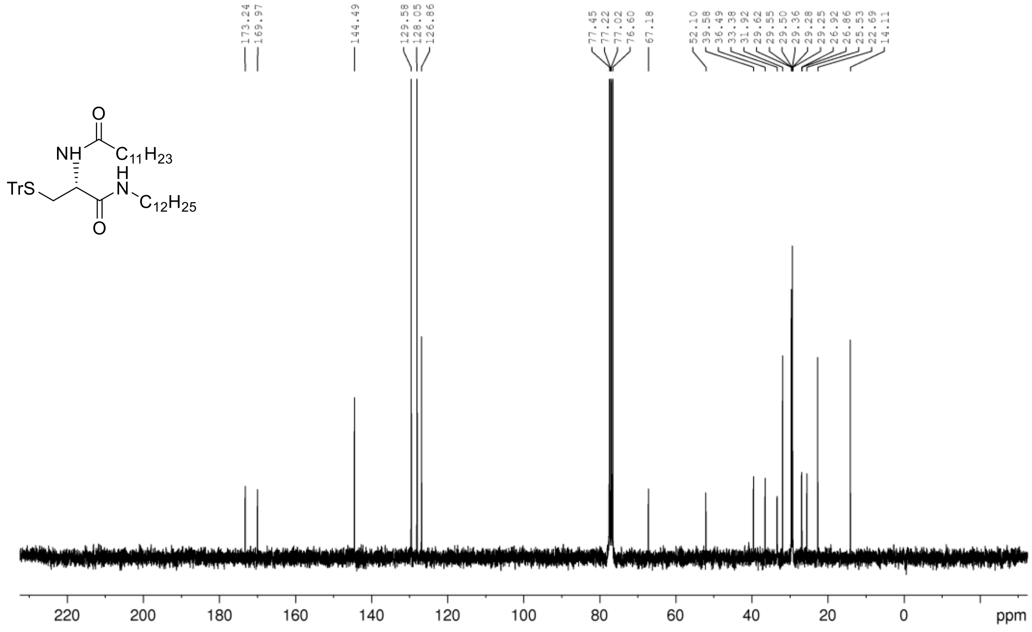

Figure S14. ^{1}H and ^{13}C NMR spectra (300 MHz, 75.5 MHz, $CDCl_3$) of **16**.

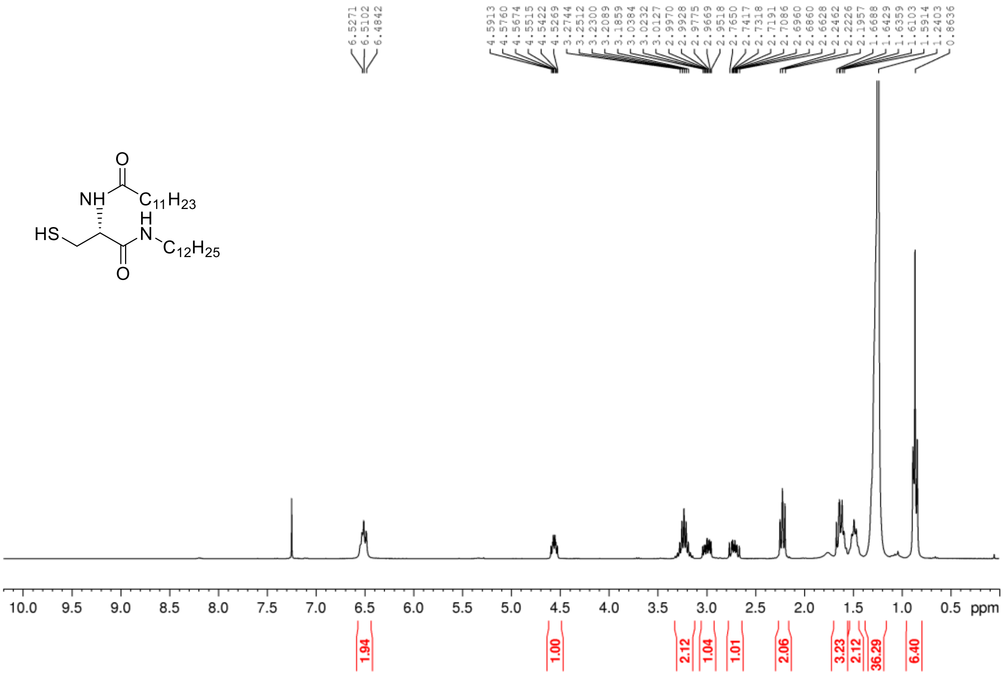

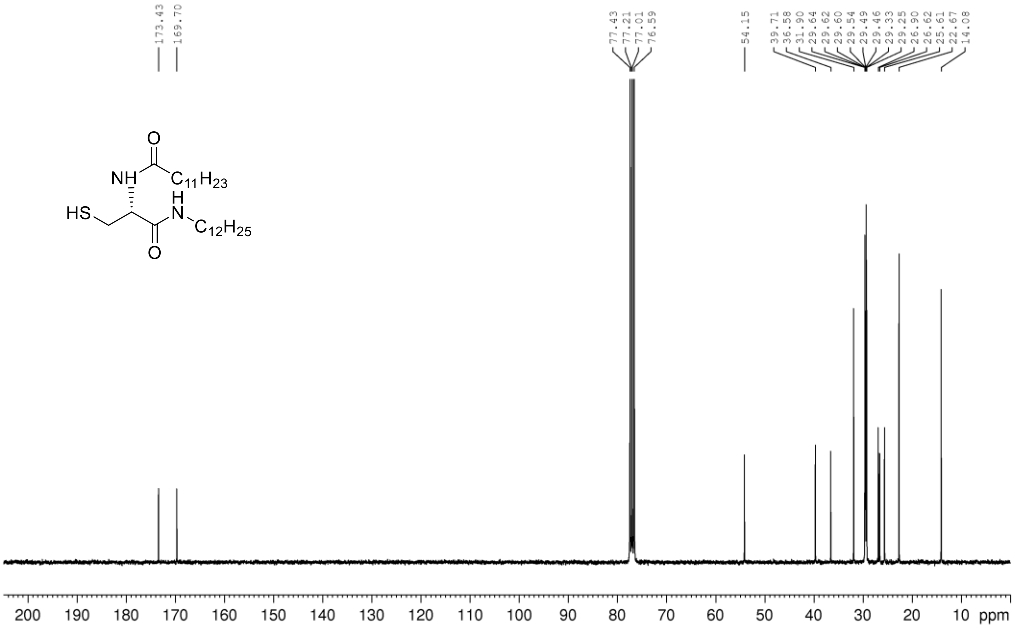

Figure S15. ^{1}H and ^{13}C NMR spectra (300 MHz, 75.5 MHz, $CDCl_3$) of **17**.

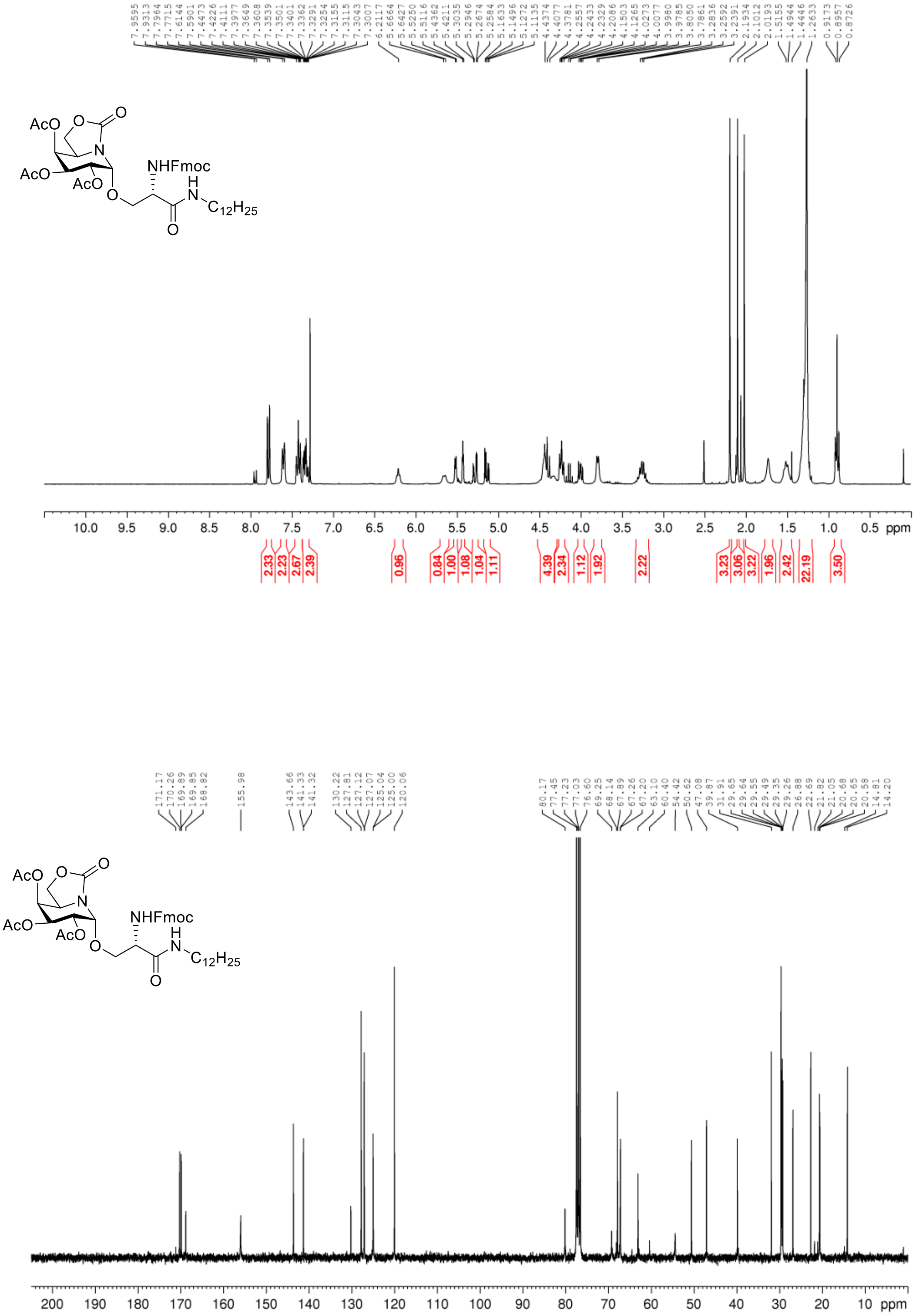

Figure S16. ^{1}H and ^{13}C NMR spectra (300 MHz, 75.5 MHz, $CDCl_3$) of **18**.

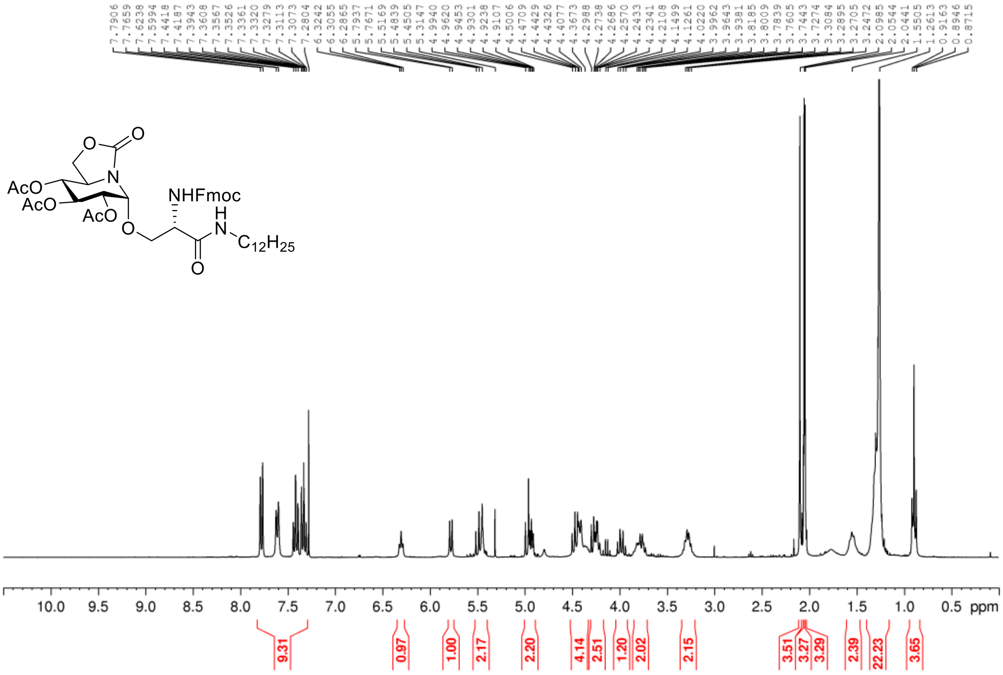

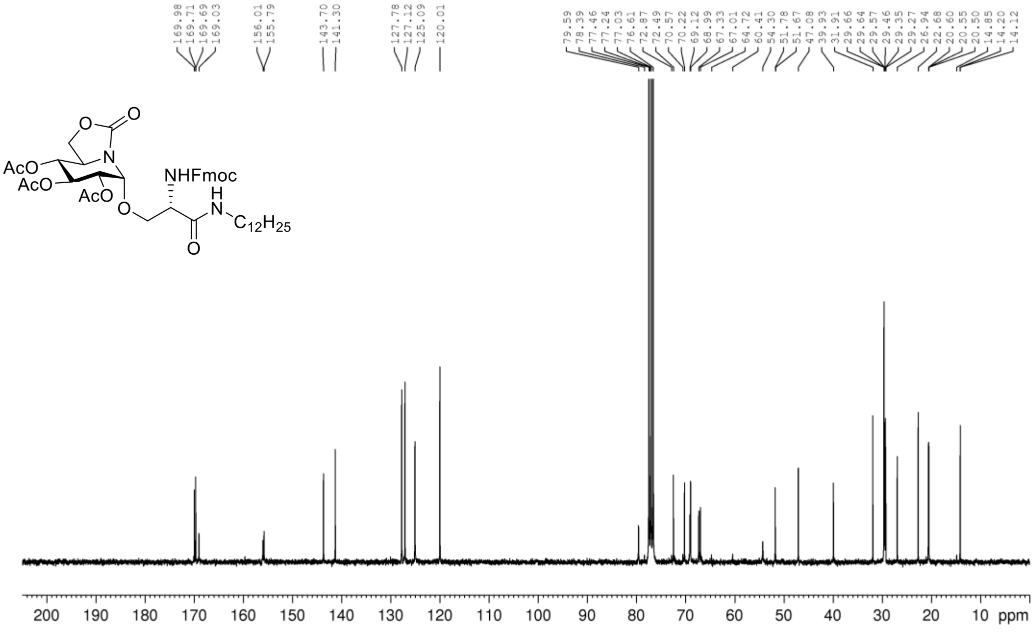

Figure S17. ^{1}H and ^{13}C NMR spectra (300 MHz, 75.5 MHz, $CDCl_3$) of **19**.

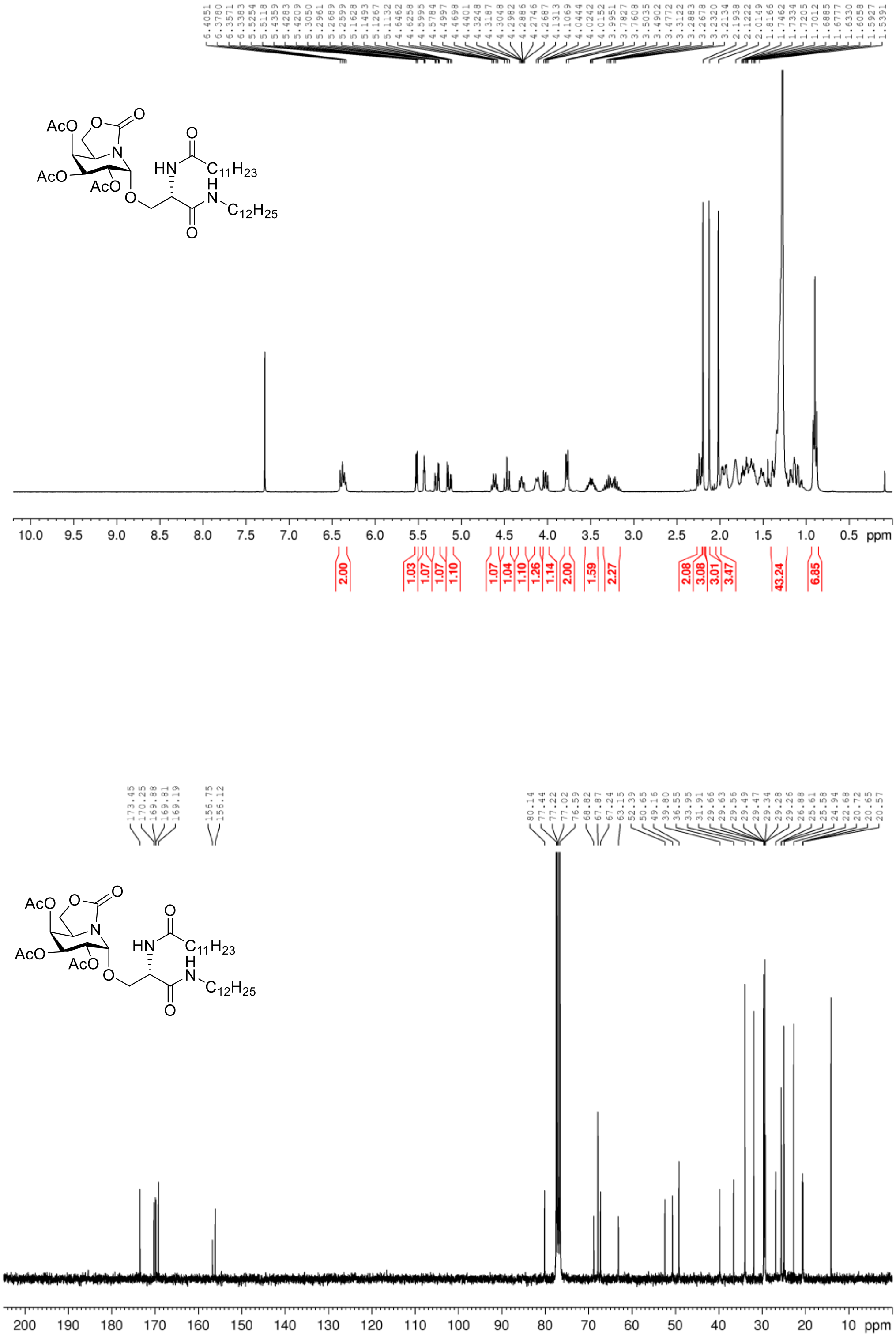

Figure S18. ^{1}H and ^{13}C NMR spectra (300 MHz, 75.5 MHz, $CDCl_3$) of **20**.

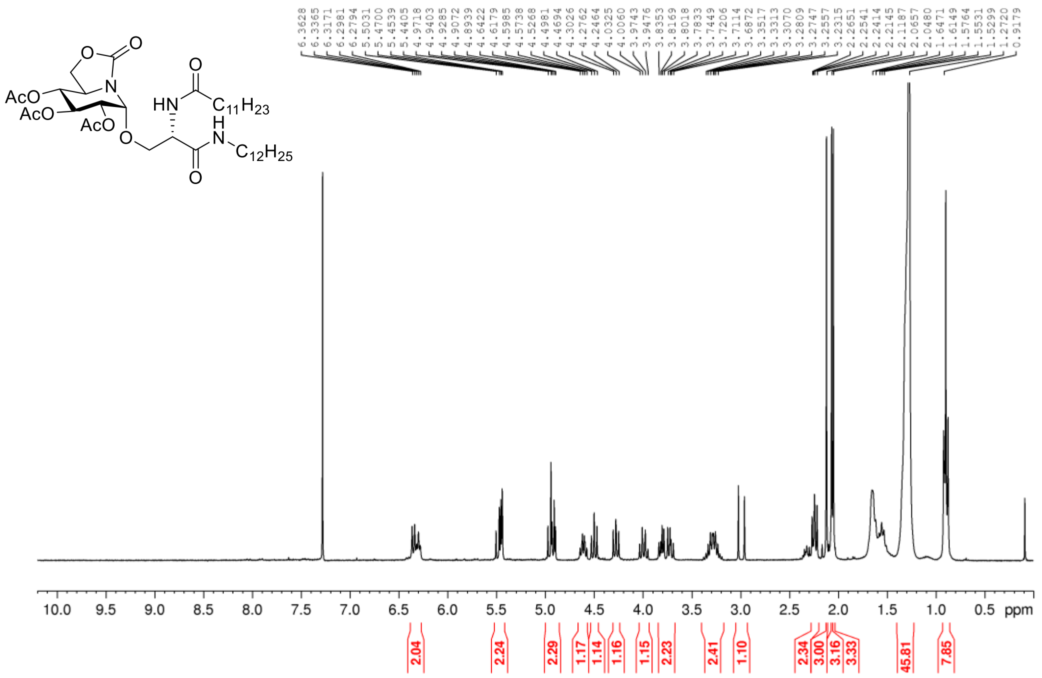

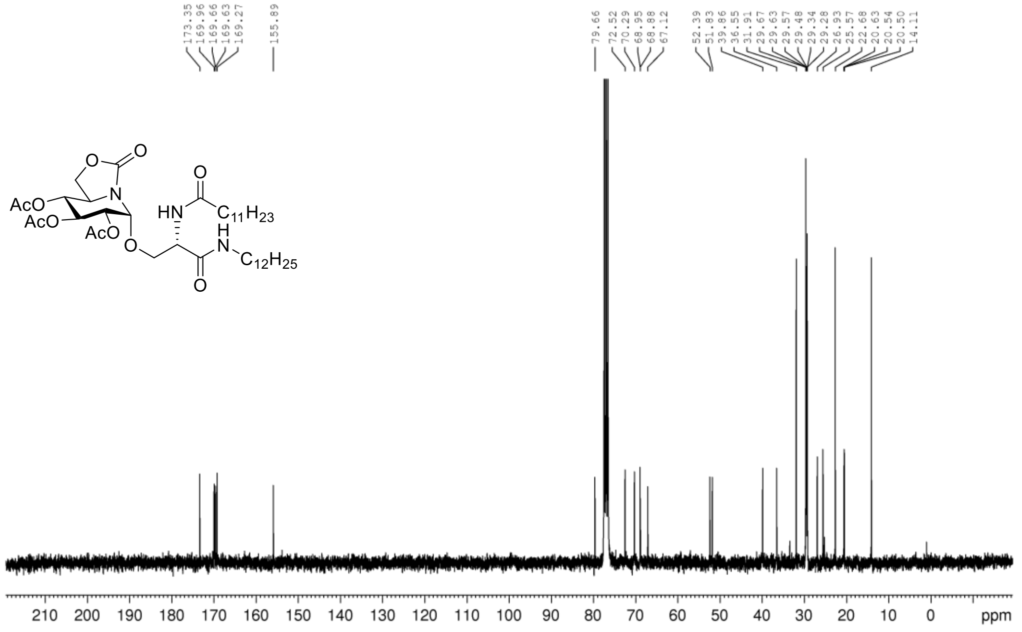

Figure S19. ^{1}H and ^{13}C NMR spectra (300 MHz, 75.5 MHz, $CDCl_3$) of **21**.

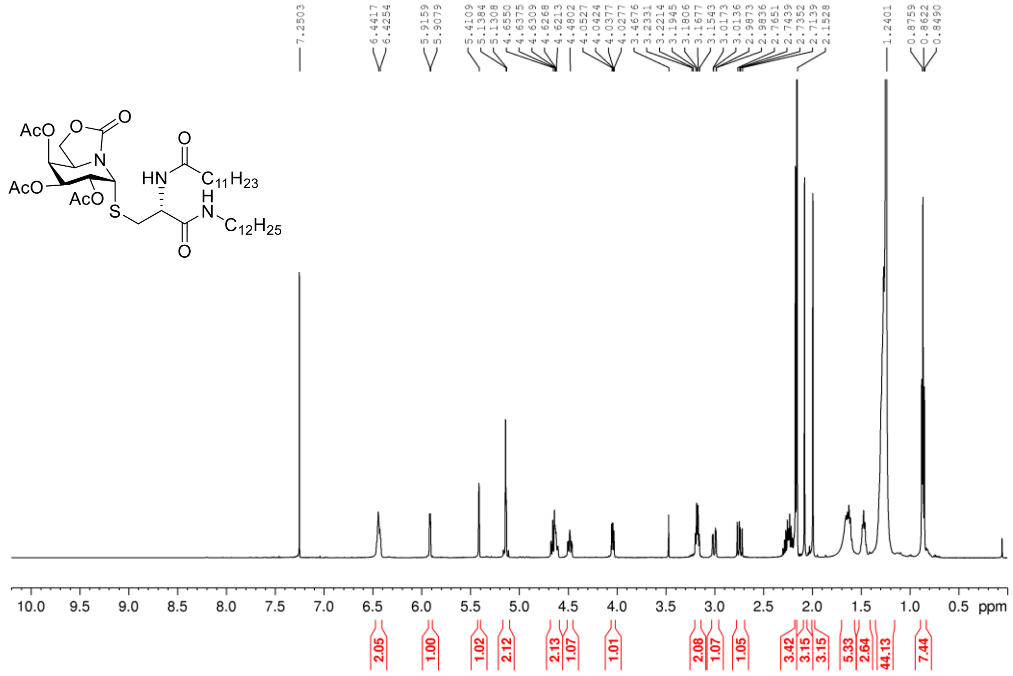

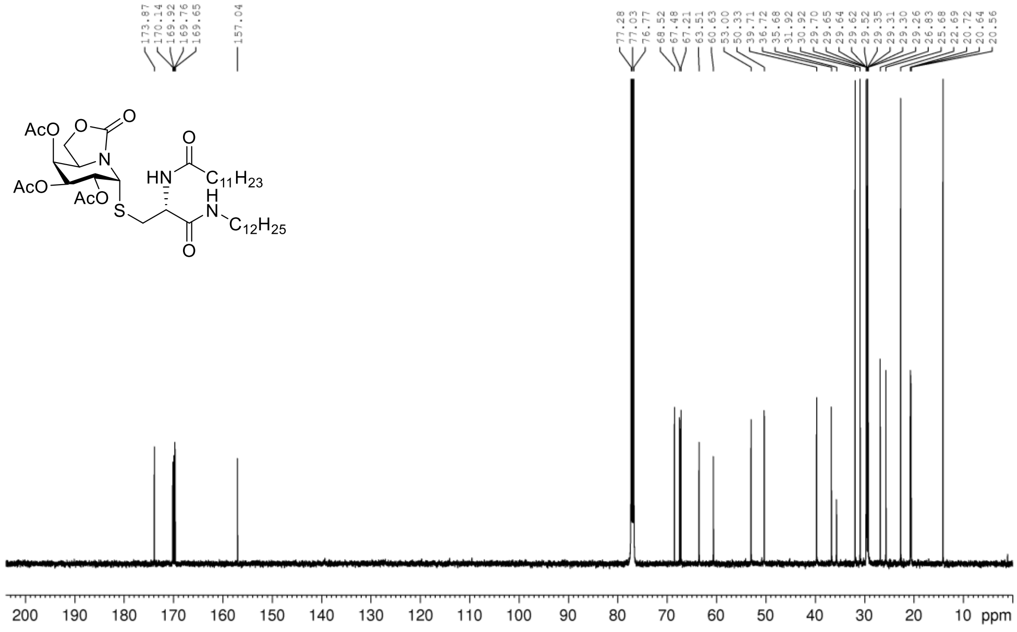

Figure S20. ^{1}H and ^{13}C NMR spectra (500 MHz, 125.7 MHz, $CDCl_3$) of **22**.

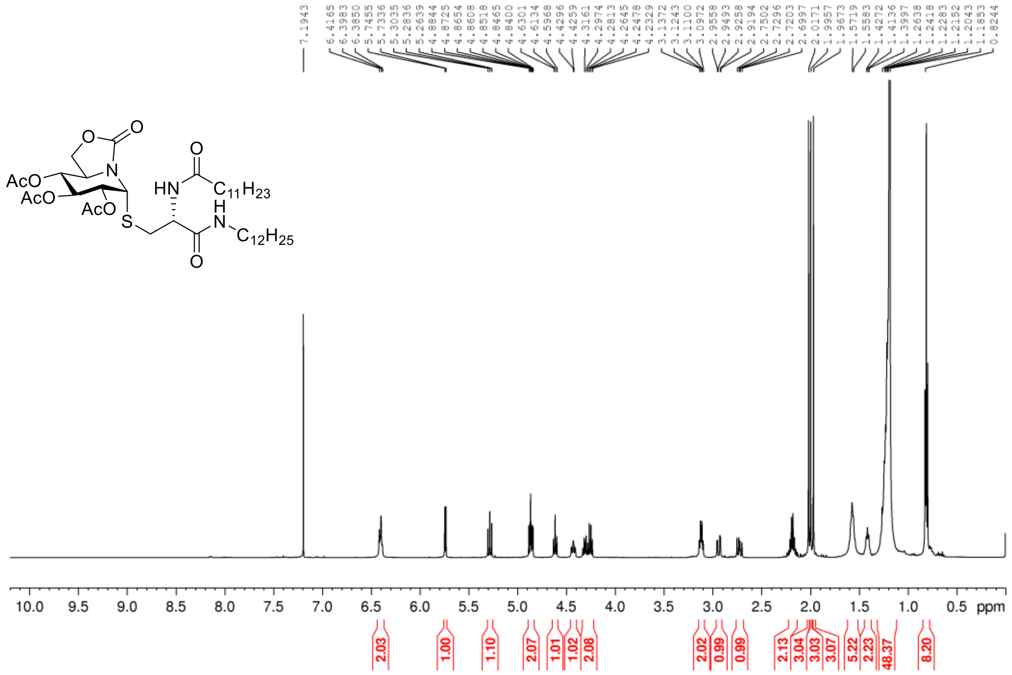

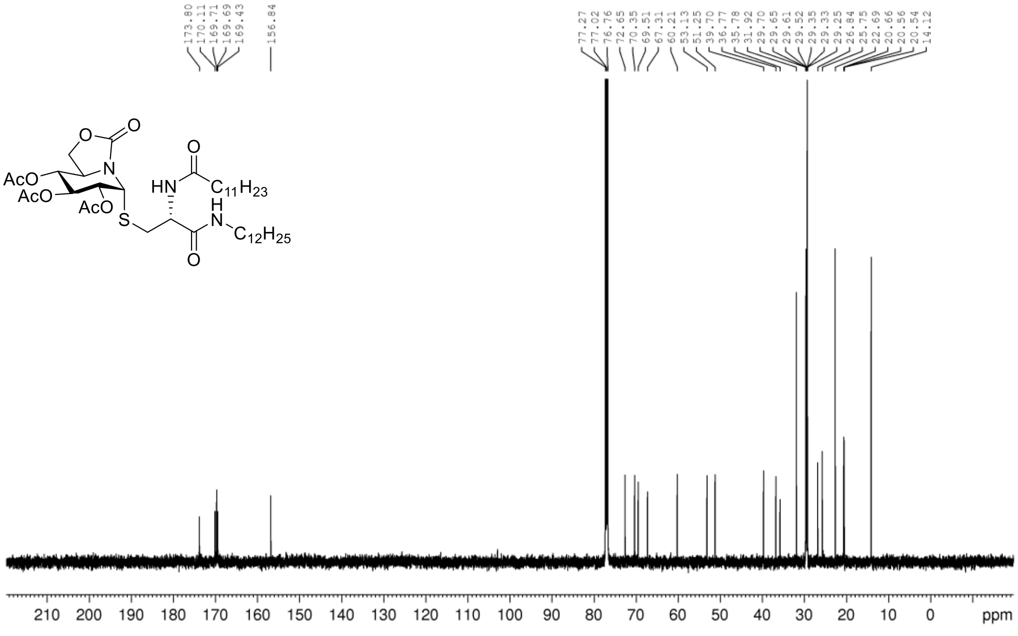

Figure S21. ^{1}H and ^{13}C NMR spectra (500 MHz, 125.7 MHz, $CDCl_3$) of **23**.

HPLC traces of final compounds

Compound 1

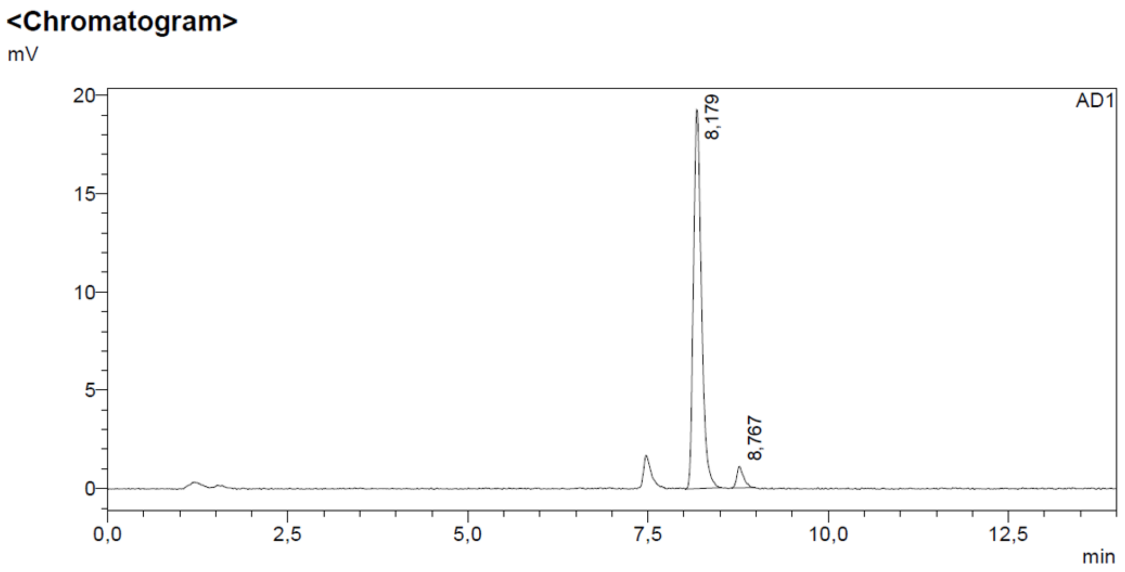

AD1

Peak#	Ret. Time	Area	Height	Area%
1	8,179	148713	19280	95,439
2	8,767	7107	1089	4,561
Total		155820	20369	100,000

Compound 2

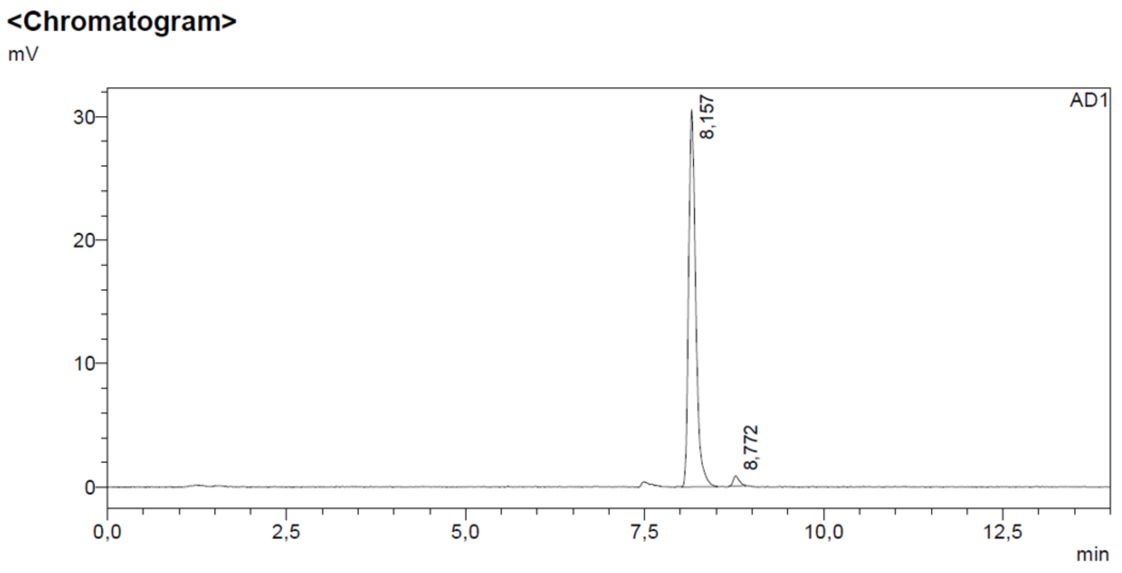

Peak#	Ret. Time	Area	Height	Area%
1	8,157	210187	30566	97,771
2	8,772	4792	820	2,229
Total		214979	31386	100,000

Compound 3

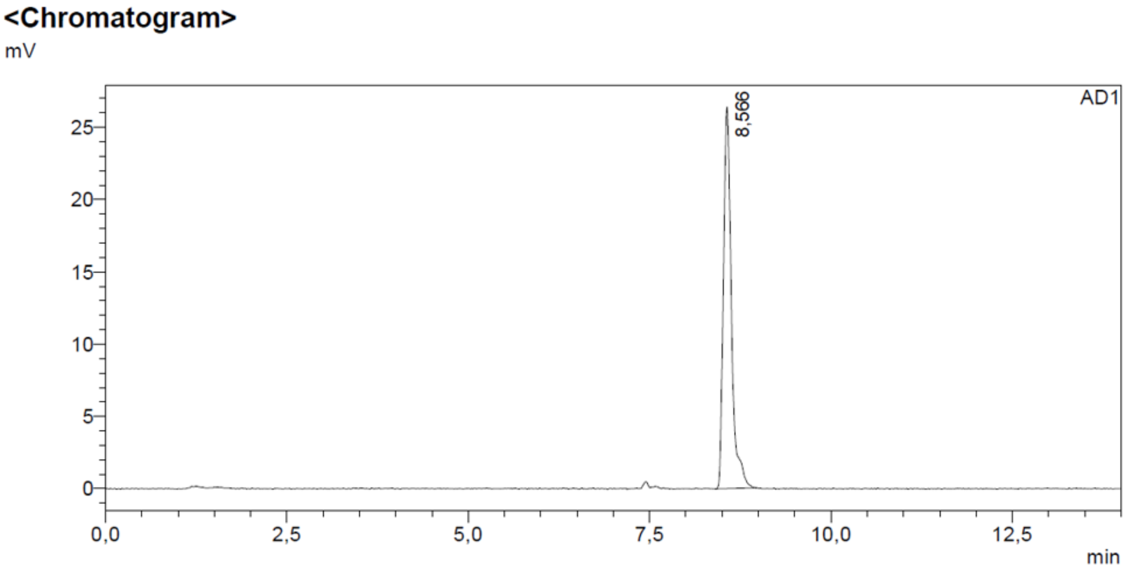

Peak#	Ret. Time	Area	Height	Area%
1	8,566	202641	26406	100,000
Total		202641	26406	100,000

Compound 4

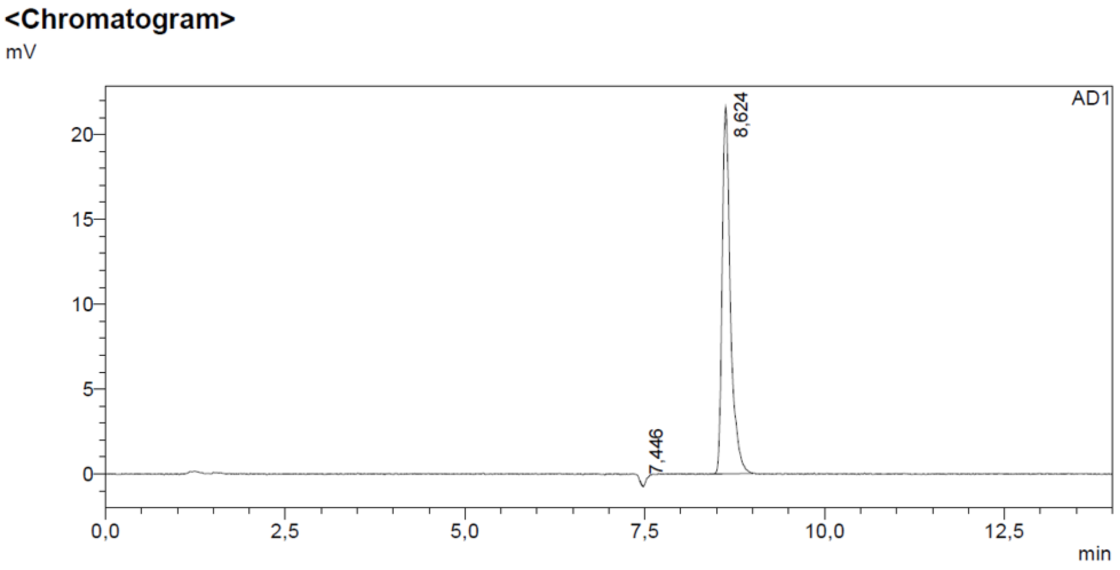

Peak#	Ret. Time	Area	Height	Area%
1	7,446	-124	-1	-0,071
2	8,624	173477	21602	100,071
Total		173354	21601	100,000
